# Type 3 porous liquids based on non-ionic liquid phases – a broad and tailorable platform of selective, fluid gas sorbents†

**DOI:** 10.1039/c9sc05770f

**Published:** 2020-01-09

**Authors:** John Cahir, Min Ying Tsang, Beibei Lai, David Hughes, M. Ashraf Alam, Johan Jacquemin, David Rooney, Stuart L. James

**Affiliations:** School of Chemistry and Chemical Engineering, Queen's University Belfast David Keir Building Stranmillis Road Belfast BT9 5AG UK s.james@qub.ac.uk; School of Physics, HH Wills Physics Laboratory, University of Bristol Tyndall Avenue Bristol BS8 5AG UK

## Abstract

We describe a series of Type 3 porous liquids, denoted “T3PLs”, based on a wide range of microporous solids including MOFs, zeolites and a porous organic polymer (PAF-1). These solids are dispersed in various non-ionic liquid phases (including silicone oils, triglyceride oils, and polyethylene glycols) which have a range of structures and properties, and that are in many cases sterically excluded from the pores of the solids. Several stable dispersions with high gas uptakes are obtained. We show how these dispersions can be tailored toward important gas separation processes (CO_2_/CH_4_, C_2_H_4_/C_2_H_6_) and applications that require biocompatibility.

## Abstract 2

We describe a series of Type 3 porous liquids, denoted “T3PLs”, based on a wide range of microporous solids including MOFs, zeolites and a porous organic polymer (PAF-1).

## Graphical abstract

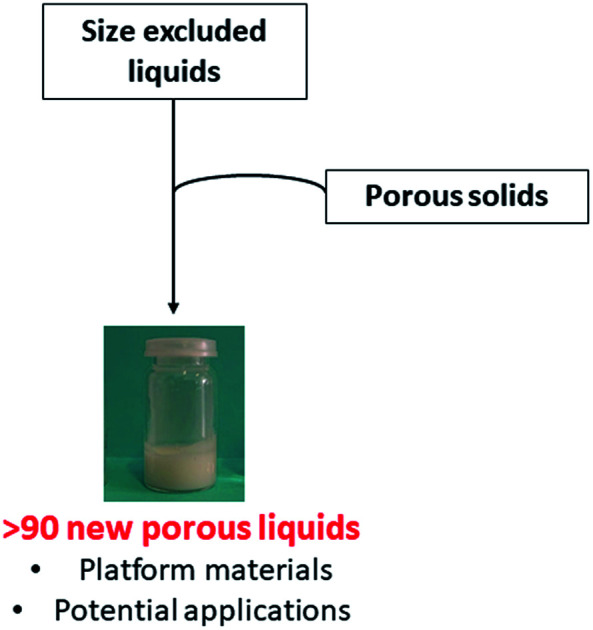

## Introduction

Porous liquids (PLs) are a new class of materials that combine the permanent, well-defined porosity of microporous solids with the fluidity of liquids.^1–4^ Their permanent porosity increases gas solubility and can engender size- and shape-selective gas dissolution, which is not possible for conventional liquids. The fluidity of PLs can also present advantages over solid sorbents. For example, being fluids, they can be engineered into continuous flow liquid-based separation processes. Important examples of current industrial gas separations based on conventional (non-porous) liquid sorbents and that are relevant to this work are the aqueous amine or polyethylene glycol-based systems for separating CO_2_ and H_2_S from methane in natural gas and biogas (gas sweetening).

Three generic types of PLs were envisaged when porous liquids were first proposed.^1^ Type 1 are discrete molecular hosts in neat liquid form, Type 2 are discrete molecular hosts dissolved in solvents which are too bulky to enter the host cavities, and Type 3 consist of particles of microporous solids dispersed in liquids that are too bulky to enter the pores of the solid. Types 1 and 2 have been reported, based on either hollow silica nanospheres, hollow carbon spheres or organic cages as the pore-defining components.^2^ Compared to Types 1 and 2, Type 3 PLs (here denoted “T3PLs”) are potentially more simply prepared in a single step from existing commercially available porous solids and liquid phases, and so currently offer the most economical preparation of the three Types. Also, the large number of possible solid–liquid combinations could make T3PLs a broad and tuneable platform of sorptive fluids with properties (such as gas uptake and selectivity, thermal/chemical stability, pore size, biocompatibility, viscosity, volatility, presence of functional groups, *etc.*) that can be easily tailored toward specific applications. T3PLs have recently been reported based on dispersions of MOFs (ZIF-8, HKUST-1) or zeolites (ZSM-5) in ionic liquids,^3^ and as glycol-based slurries.^4^ Increased gas uptake in these dispersions compared to the neat liquid phases has been demonstrated. However, examples based on non-ionic liquid phases remain few.^4^ Given the huge range of porous solids and non-ionic liquid components from which T3PLs can be comprised, it is clear that full scope of these electrically neutral phases remains unexplored.

Here, we describe work that points to the greater breadth of this class of materials. Specifically, we show that a wide range of microporous solids, (MOFs, zeolites, PAF-1) can be dispersed into diverse chemically inert and thermally stable non-ionic liquid phases (silicone oils, polyethylene glycols, naturally occurring triglyceride oils such as olive oil, castor oil, *etc.*) that are sterically excluded from the pores of the solid. In many cases, stable dispersions are obtained simply by prolonged magnetic stirring and with little or even no chemical modification required to the surface of the solid phase. Many such dispersions exhibit high, selective and predictable levels of gas uptake. Capitalising on the diversity of these designable materials, we describe examples that have been tailored toward significant large scale gas separation challenges, specifically CO_2_/CH_4_ separation (relevant to natural gas and biogas sweetening) and ethene/ethane separation. We also describe a T3PL which is, in principle, edible, suggesting potential for applications where biocompatibility is important.

## Results and discussion

### Rationale

**Fig. 1:**
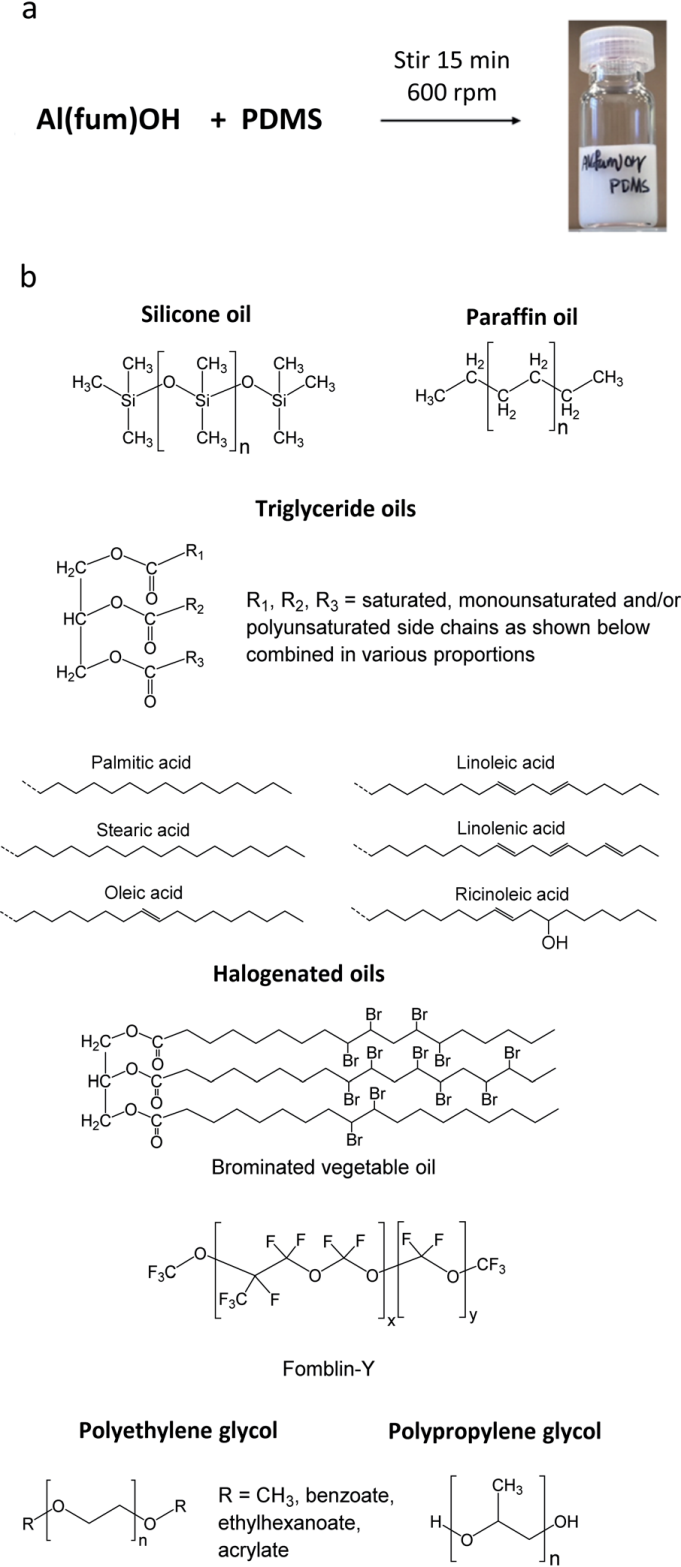
(a) An example of a Type 3 porous liquid (T3PL) preparation from Al(fum)(OH) and PDMS (see ESI 3†); (b) the range of liquid phases chosen for this study (see ESI 1†).

The porous materials and liquid phases explored in this study are indicated in Fig. 1 and Tables 1 and 2. The solids include some of the archetypal and widely available MOFs and zeolites. Notable features include the presence of open metal sites (HKUST-1), high chemical and thermal stabilities (ZIF-8, UiO-66, UiO-67, MOF-801, Al(fum)(OH), MIL-53(Al), CAU-10, zeolites) and biocompatibility (CD-MOF-1). A purely organic porous material (PAF-1) is also included in this study. The liquid components explored (Fig. 1) were selected based on their likelihood of being sterically excluded from the pores of the solids, their availability, and the ease with which parameters such as viscosity, density and biocompatibility could be varied.

**Table 1:** Summary of Type 3 PLs (T3PLs) formed by dispersion of microporous solids in PDMS, halogenated oil and paraffin oil liquid phases^a^

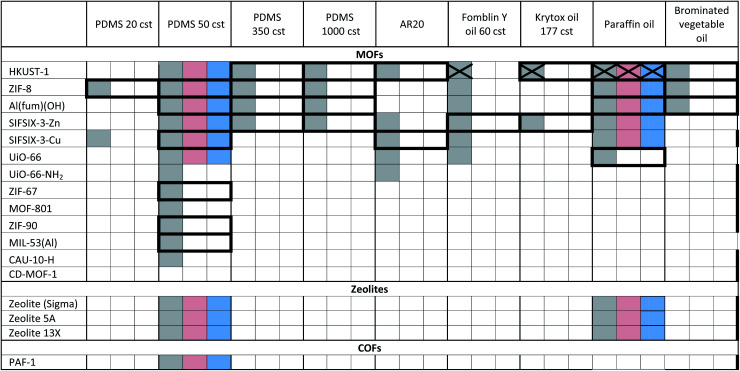

a Key: bold borders indicate dispersions that are stable to sedimentation and flotation for at least 1 day visually. Filled boxes indicate solid–liquid compositions that have been investigated in this work. 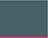: enhanced CO_2_ solubility observed; 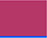: enhanced CH_4_ solubility observed; 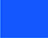: enhanced N_2_ solubility observed; 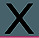: no enhanced CO_2_ uptake observed.

**Table 2:** Summary of Type 3 PLs (T3PLs) formed by dispersion of microporous solids in triglyceride oils and polyethylene glycol derivative liquid phases^a^

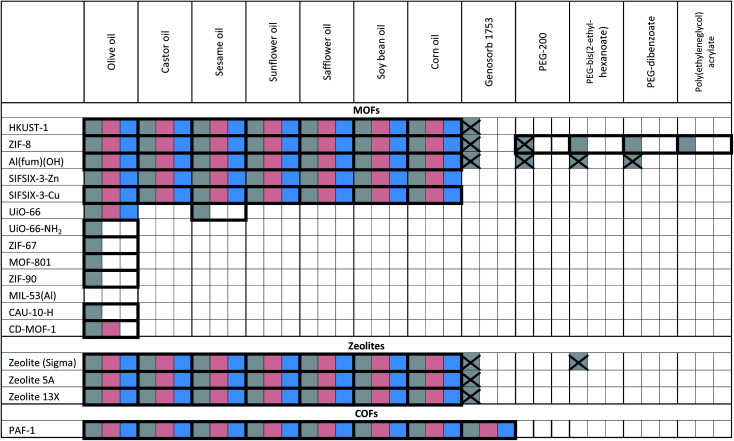

a Key: bold borders indicate dispersions that are stable to sedimentation and flotation for at least 1 day visually. Filled boxes indicate solid–liquid compositions that have been investigated in this work. 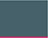: enhanced CO_2_ solubility observed; 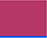: enhanced CH_4_ solubility observed; 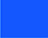: enhanced N_2_ solubility observed; 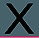: no enhanced CO_2_ uptake observed.

For example, silicone oils have bulky SiMe_3_ end groups that should be sterically excluded from the pores of the solids, are widely available in various chain lengths to provide a range of viscosities and are biocompatible. Triglyceride oils have branched core structures that should sterically prevent their inclusion into the pores of the solids, are naturally occurring, biodegradable, biocompatible and widely available with a range of side chains. Triglyceride oils are known to break down to trans fats, however this is unlikely to happen at temperatures in which the porous liquids are likely to be exposed (<323 K) (ESI 1†).^5^ Halogenated oils such as brominated vegetable oil and the fluorinated oil Fomblin® Y, have high densities (1.33 g cm^−3^ and 1.8–1.9 g cm^−3^ respectively) which could be useful for obtaining stable dispersions with high-density solids. Polyethylene glycol (PEG) derivatives are relatively polar and strongly coordinating due to their ether functionalities. They are widely available in liquid forms with a range of viscosities, and with various end groups, some of which may be bulky enough to be sterically excluded from the pores of the solids. Included in this study is Genosorb® 1753 which consists of a mixture of polyethylene glycol dimethylethers, CH_3_O(CH_2_CH_2_O)_*n*_CH_3_ (*n* ∼ 4–10), and is an import physical solvent used in gas purification including natural gas sweetening.^6^

### Formation of dispersions and their stabilities

**Fig. 2:**
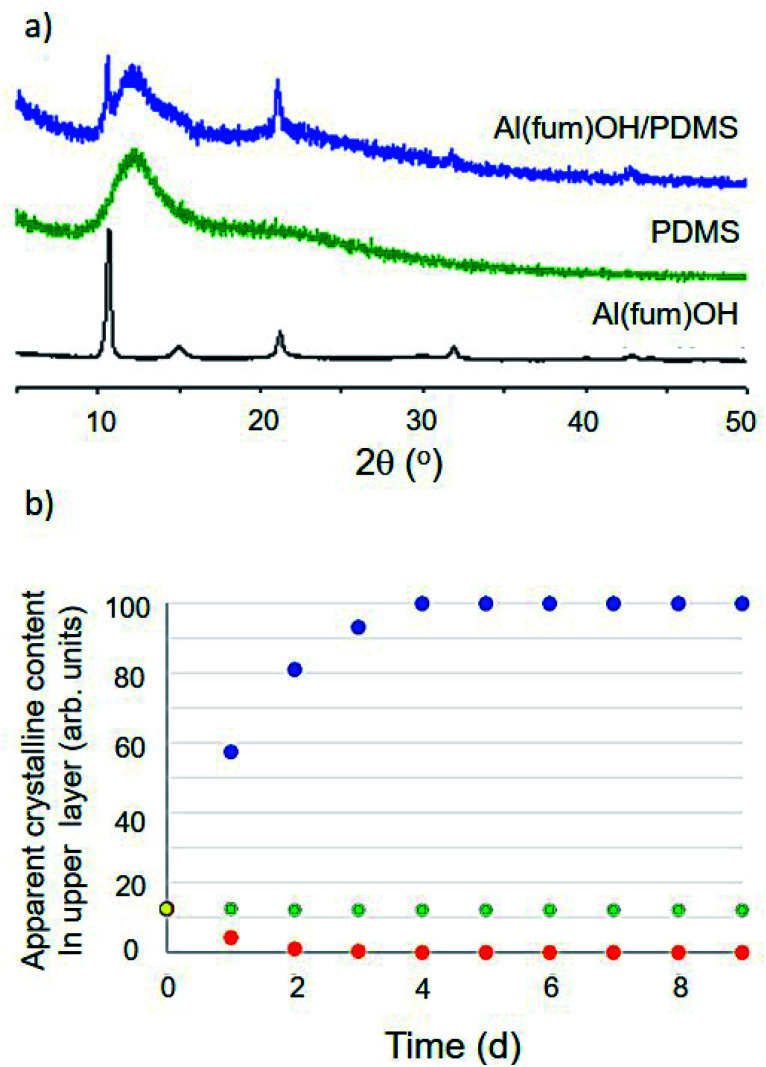
(a) Comparison of PXRD patterns for the solid MOF Al(fum)(OH), liquid PDMS and the Al(fum)(OH)/PDMS porous liquid dispersion showing that the pattern for the dispersion is a simple superposition of the patterns of its two components. (b) Analysis of dispersion stability over 9 days, by PXRD of the top portion of the porous liquid, illustrating three scenarios: (i) sample 1 (Al(fum)(OH)/brominated vegetable oil), solid floats to the surface, blue series, (ii) sample 2 (Al(fum)(OH)/PDMS), the solid component sediments out, red series, and (iii) sample 3 (ZIF-8/PDMS), the dispersion is stable over at least 9 days, green series (ESI 5 Fig. S14†).

Initial conditions used to form dispersions involved simple magnetic stirring for 15 minutes to 2 hours or ball milling for 2–10 minutes (Fig. 1a and ESI 3†). The obtained dispersions were analysed initially by PXRD to confirm that the porous material remained intact (Fig. 2 and ESI 4†). PXRD patterns consisted in all cases of a superposition of the patterns of the two components, *i.e.* peaks due to the porous solid superimposed upon broad features due to the liquid, confirming that the solids retained their structures and crystallinity.

Notably, in the initial scoping study, with regard to the solid component, no precautions were taken as to the particle size or surface functionality, *i.e.* the porous solids were used directly from standard synthetic methods. Even so, as shown in Tables 1 and 2, in many cases stable dispersions (*e.g.* ZIF-8/PDMS and HKUST-1/triglyceride oils) were obtained (ESI 5, Fig. S14†) from this simple preparative method. Following this positive screening, we explored how the stabilities of selected dispersions could be increased further by various methods.^7^ Specifically: (i) Reducing the MOF particle size (from 400 nm to <50 nm) by ball milling or rapid crystallisation from solution increased the stability of HKUST-1/PDMS dispersions (from <1 d to 3 d), as well as Al(fum)OH/PDMS dispersions (from <1 d to 30 d) respectively (ESI 5, Fig. S7 and S8†), (ii) increasing the attractive interactions between the MOF and liquid phase (by changing from PDMS to poly(methylphenyl)silicone (AR20)) or by functionalising the surface of HKUST-1 with silsesquioxane (OPOSS) groups,^8*a*,8*b*^ improved the stability of HKUST-1 dispersions (from <1 d to >10 d or 3 d respectively) (ESI 5, Fig. S10 and S11†), (iii) increasing the viscosity of the PDMS (from 20 cst to 1000 cst) delayed the sedimentation of HKUST-1 dispersions (from <1 d day to *ca.* 7 d) (ESI 5, Fig. S12†), (iv) increasing the density of the liquid component by using halogenated oils such as Fomblin® Y or brominated vegetable oil enabled stable dispersions to be prepared with the relatively high density fluorine-containing MOF SIFSIX-3-Zn (1.57 g cm^−3^),^9^ which did not form stable dispersions in silicone or triglyceride oils (ESI 5, Fig. S13†). These observations illustrate that through a range of intuitive approaches stability to sedimentation or flotation can be significantly increased if needed (ESI 5†).

The dispersion stability was also quantified for selected examples through PXRD analysis of the upper layer of the suspension. The ratio of crystalline (solid) to amorphous (oil) content was determined by a known application of Rietveld refinement and monitored for up to nine days (Fig. 2b and ESI 5†).^10^ Given the large number of mixtures prepared, only a selection (specifically Al(fum)(OH)/brominated vegetable oil, Al(fum)(OH)/PDMS and ZIF-8/PDMS) were analysed by this method. Fig. 2b shows data for these three dispersions, each showing different behaviour, specifically low stability due to flotation of the solid (Al(fum)(OH)/brominated vegetable oil), good stability (ZIF-8/PDMS) and low stability indicated by sedimentation (Al(fum)(OH)/PDMS).

### Gas solubilities

**Fig. 3:**
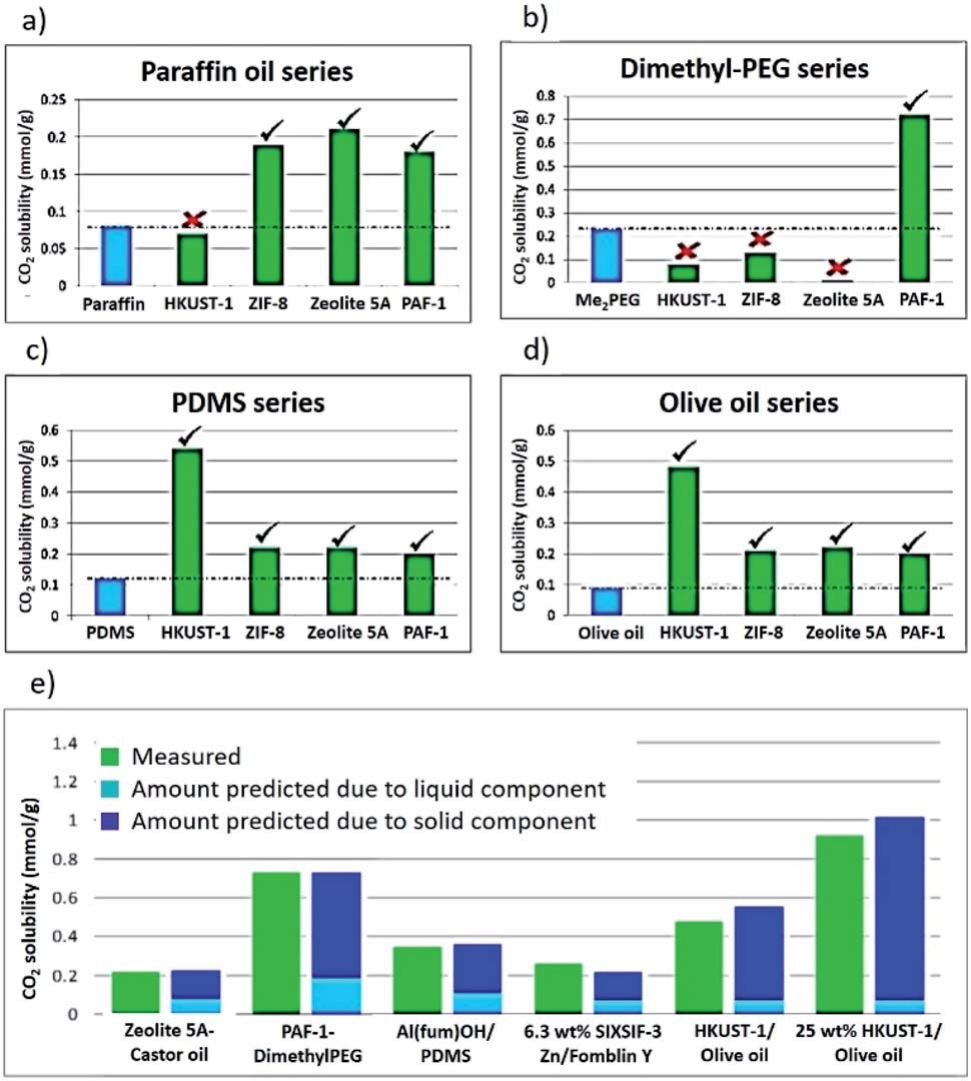
Selected examples of CO_2_ solubilities at 0.8 bar in pure liquids and corresponding porous liquids. For (a)–(d) labels below bars indicate the porous solid component added to the liquid phase. (a) Paraffin oil series; (b) dimethylether polyethylene glycol (Genosorb® 1753) series; (c) PDMS series; (d) olive oil series. (e) Comparison of predicted and measured gas solubilities in the Type-3 porous liquids. Amount of added solid is 12.5 wt% unless otherwise indicated. Ticks indicate enhanced CO_2_ solubility and therefore the existence of empty pores. Crosses indicate lower CO_2_ solubility suggesting that the pores have been filled by the liquid.

Having formed a range of dispersions, some with stabilities of days to weeks, a critical next question is whether the pores of the dispersed solids are still empty and available to guests, *i.e.* whether the molecules of the liquids do, as hoped, remain outside the pores. To probe this, we screened the solubilities of gases in these PLs using an isochoric method described elsewhere.^11^ Initially CO_2_ solubilities at *ca.* 0.8 bar in the putative porous liquids were first measured and compared to the CO_2_ solubilities in their pure liquid components (Fig. 3a–e; details in ESI 6†). A minority of the dispersions (*e.g.* HKUST-1/paraffin, ZIF-8/Genosorb® 1753) showed the same or even lower CO_2_ uptake than that observed for their neat liquid component (*i.e.* there was no enhancement of gas uptake due to the presence of the porous solid). This occurred particularly where non-bulky liquid molecules were used and we attribute this to the ability of those liquids to penetrate into the pores of the solid, but it is also possible, in principle, that coordinating species such as PEG-type polymers block gas inclusion by adhering strongly to the external surfaces of the solid particles. Supporting the inclusion hypothesis, PEG chains with bulky end groups (*i.e.* benzoate, acrylate and ethylhexanoate) did give dispersions with enhanced CO_2_ uptake (for example, see entries for ZIF-8 in Table 2). Remarkably, for most of the dispersions prepared (Table 1) the CO_2_ solubility was increased by a factor of 3–6 compared to their neat liquid components (ESI 6†). Illustrative examples include HKUST-1/PDMS and HKUST-1/olive oil (Fig. 3). As described in the Rationale above, in most cases this can be correlated with intuitive steric effects; the bulky SiMe_3_ end groups of PDMS and the branched core structure of olive oil (a triglyceride), sterically prevented them from entering the pores of MOF, leaving the pores accessible to gases. However, at least one instance of high gas uptake are surprising. In particular, PAF-1 has large pores (pore size distribution centred around 1.4 nm),^12*a*,12*b*^ which would be expected to be large enough for non-bulky linear polymers such as Genosorb® 1753 to diffuse into. However, the dispersion PAF-1/Genosorb® 1753 shows significantly enhanced CO_2_ uptake (0.72 mmol g^−1^) compared to the pure liquid (0.23 mmol g^−1^), suggesting that the full porosity of PAF-1 remains accessible when dispersed in this medium (ESI 6†) (note that MOFs with smaller pores such as HKUST-1 (0.9 nm), and Al(fum)OH (0.6 nm) do appear to become occupied by Genosorb® 1753 (Table 2)).

In the above studies the amount of porous solid used was arbitrarily chosen to be 12.5 wt%. However, we noted that for some of the most stable dispersions, such as HKUST-1/olive oil, it was possible to increase this to 25 wt% without noticeable sedimentation.

Comparison of CO_2_ uptake using silicone oils of different viscosities revealed that there was no difference in the amount of gas uptake, but that the rate of uptake was slower for the more viscous oil as expected (ESI 7†).^13^

### Positron annihilation lifetime spectroscopy (PALS)

**Fig. 4:**
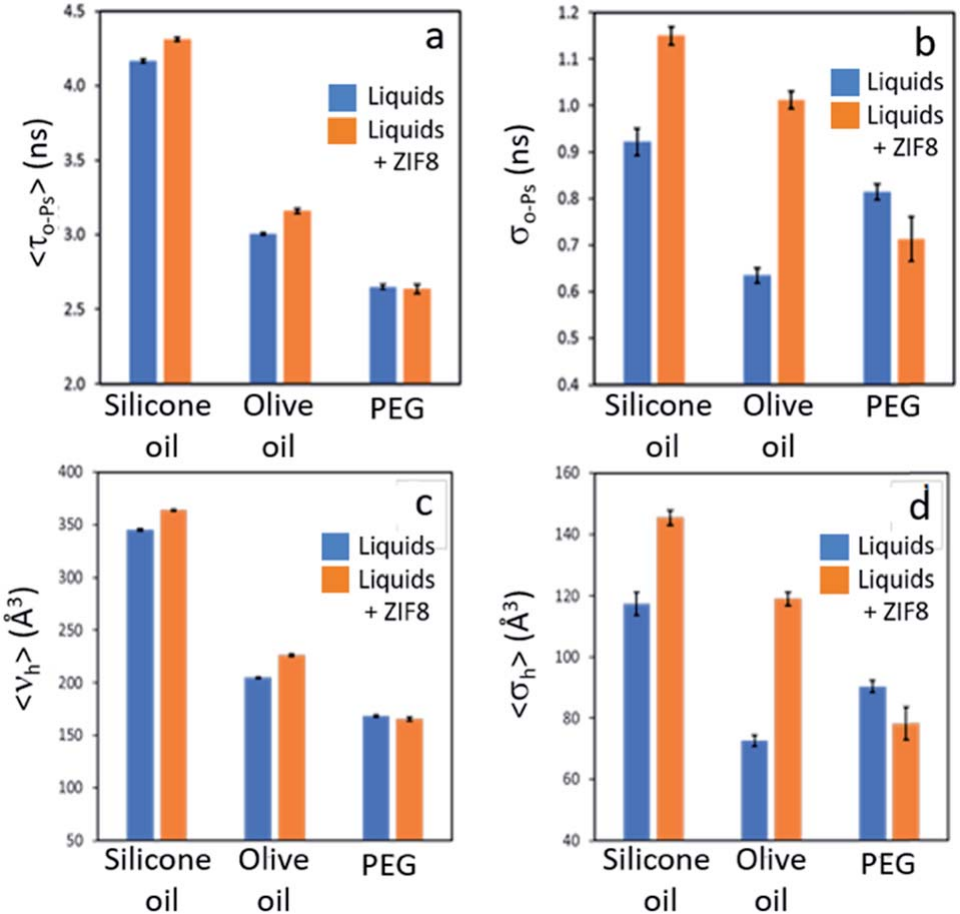
Porosity size/size-distribution derived from PALS for neat liquids silicone oil, olive oil, and polyethylene glycol, (PEG) *vs.* ZIF-8/liquid dispersions in those liquids: (a) 〈*τ*_*o*-Ps_〉> – average *o*-Ps lifetime, a measure of the average pore size; (b) *σ*_*o*-Ps_ – width of *o*-Ps lifetime distribution indicating a spread around 〈*τ*_*o*-Ps_〉; (c) 〈*v*_h_〉 – average pore volume derived from 〈*τ*_*o*-Ps_〉; (d) *σ*_h_ – spread of pore volume around 〈*v*_h_〉.

The permanent porosity of selected dispersions was further supported by positron annihilation lifetime spectroscopy (PALS).^14,15^ Addition of 12.5 wt% ZIF-8 to silicone oil or olive oil resulted in appreciable increases in the average pore sizes together with remarkably larger increases in the pore size distributions (Fig. 4; details in ESI 8†) compared to the neat liquids. This indicates the presence of the empty pores in the porous liquid. In contrast, dispersion of ZIF-8 into polyethylene glycol (PEG, with non-bulky OH end groups) did not increase either the average pore size or the pore size distribution, indicating that the ZIF-8 pores are occupied by PEG. This observation correlates well with the CO_2_ solubilities measured for these PLs (Tables 1 and 2).

### Tailoring T3PLs toward applications

A significant point is that in all cases where increased gas uptake was seen, the amount of uptake was, within error, equal to the weighted average of the gas uptakes of the pure solid and liquid components (ESI 6†). Similar observations have been made for ionic-liquid based T3PLs.^3,4^ This means that gas uptakes in dispersions are, therefore, predictable, facilitating rational design. As a simple example, by increasing the amount of the HKUST-1 component to 25 wt%, the estimated total pore volume of HKUST-1/olive oil increases from 7.1% to 14.3% and the CO_2_ solubility increased correspondingly from 0.5 to 0.9 mmol g^−1^ (Fig. 3e).

For applications in cyclic gas separations, ease of removing the dissolved gas (regeneration) is critical. It is well-established that a major drawback of amine-based CO_2_ scrubbing technology is the large energy cost of regenerating the amine solution. In the above studies, the solid component was activated before being combined with the liquid. It was notable, therefore, that due to the high thermal stability of PDMS, it was also possible to activate (*i.e.* remove guests included during the synthesis) the HKUST-1/PDMS porous liquids after formation of the dispersions, using the same activation conditions (200 °C, 2 h) as for the pure MOF component. This supports the possibility that T3PLs might also be easily regenerated *in situ*. PDMS polymers with high molecular weights have low vapour pressures (ESI 1†), and so evaporation of the liquid should not be an issue.^16^ In general (ESI 1†), it can be noted that vapour pressures for the liquids used here are in most cases lower than that of Genosorb® which is already used as a solvent for natural gas sweetening. Also, for triglyceride oils, their smoke points (160–310 °C) are high enough for decomposition to be avoided during regeneration (typically <100 °C). We further studied the regeneration of a 12.5 wt% HKUST-1/PDMS porous liquid with regard to CO_2_ uptake and removal. As can be seen in Fig. S20,† exposure to reduced pressure (≤8 × 10^−2^ bar, 30 minutes) was sufficient to recover at least 90% of the PL capacity. By contrast, under the same conditions a 12.5 wt% aqueous amine solution showed little or no regeneration. This difference can be expected because CO_2_ is bound relatively weakly in the MOF pores through physical interactions rather than through strong chemical bonding found in the amine systems (details in ESI 9†).

**Fig. 5:**
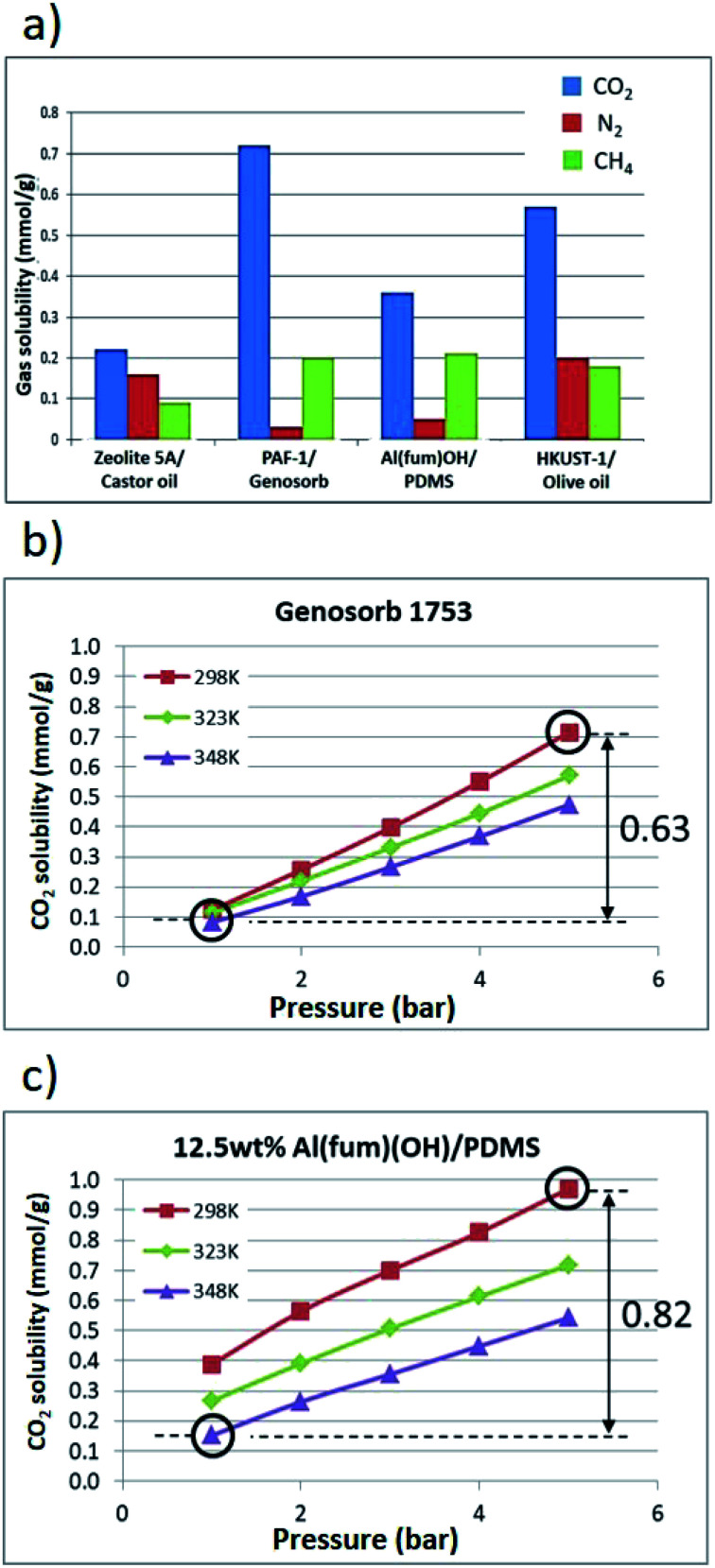
(a) Comparison of CO_2_, N_2_ and CH_4_ solubilities in selected T3PLs illustrating generally selectivity for CO_2_, (b) CO_2_ solubilities in Genosorb® 1753 from 1–5 bar at 298 K, 323 K and 348 K, (c) CO_2_ solubilities in 12.5 wt% Al(fum)(OH)/PDMS from 1–5 bar at 298 K, 323 K and 348 K. The implied CO_2_ working capacities under temperature and pressure swing conditions are indicated by double-headed arrows.

Further gas solubility studies generally revealed significantly lower CH_4_ and N_2_ uptake compared to CO_2_ for these T3PLs, suggesting the possibility to apply them in separation of CO_2_ and CH_4_ (Fig. 5a and ESI 6, Tables S3–S7†). Al(fum)(OH) was selected for further studies due to its good CO_2_ uptake as well as its high physicochemical stability. To form a porous liquid it was combined with low viscosity PDMS (50 cst) to form Al(fum)(OH)/PDMS. As a benchmark for comparison, we selected Genosorb® 1753, because of its use as a physical solvent in separating CO_2_ and from natural gas (natural gas sweetening).^17^ Gas separations based on liquid solvents can potentially operate under temperature and/or pressure swing conditions.^18^ We therefore compared CO_2_ solubilities in Al(fum)(OH)/PDMS and Genosorb® 1753 over a range of pressures (1–5 bar) and temperatures (298 K, 323 K and 348 K). Encouragingly, as shown in Fig. 5b and c, 12.5 wt% Al(fum)(OH)/PDMS not only has greater CO_2_ capacity at low pressure (0.39 mmol g^−1^*vs.* 0.12 mmol g^−1^), but also a greater working capacity than Genosorb® 1753 (0.82 mmol g^−1^*vs.* 0.63 mmol g^−1^) under these simulated temperature and pressure swing conditions (*i.e.* when operating between 348 K, 5 bar and 298 K, 1 bar). It is apparent from Fig. 5c that the slopes of the uptake curves for the porous liquid are greater between 1–2 bar than above 2 bar. This corresponds to the majority of the enhanced uptake due to the Al(fum)OH occurring at these low pressures, which is as expected from the uptake for the pure solid. Although many other factors need to be taken into account before considering applications, this does suggest that T3PLs can be engineered to out-perform conventional non-porous solvents used in gas separations in terms of usable capacity.

An exciting further possibility is that T3PLs could be applied to gas separations which are currently not amenable to liquid-based processes because of the lack of a suitably selective solvent. For example, ethane and ethene are currently separated by cryogenic distillation, but the high energy cost of this process makes alternative approaches attractive.^16^ Conventional solvents do not show high selectivity for either gas over the other.^18^ Silver(i)- and copper(i)-containing liquids including ionic liquids (Ag-ILs) have been found to have high ethene selectivity.^19^ Porous solids including zeolite AgA have also been investigated for this separation, but solids cannot be circulated in continuous flow processes.^20^ A T3PL was therefore formulated based on a 12.5 wt% dispersion of zeolite AgA (to engender selectivity for ethene over ethane), in paraffin oil (as a simple, economical and stable liquid phase, that is expected to be size-excluded from zeolite AgA).^21^ As hoped, the AgA/paraffin oil T3PL was selective toward ethene over ethane by a factor of approximately 4.5 (solubility of ethene at 0.8 bar, 25 °C, 0.35 mmol g^−1^; solubility of ethane, 0.08 mmol g^−1^; 0.8 bar, 25 °C) (ESI 11, Tables S9–S11†). Although the pure paraffin solvent preferentially dissolves ethane over ethene by a factor of *ca.* 2 (ESI 11†), the capacity and selectivity of the zeolite AgA for ethene dominates in the porous liquid.

To further explore the applicability of T3PLs in other general areas, we sought to prepare an example that was biocompatible. Specifically, we attempted to form dispersions of CD-MOF-1,^22^ a biocompatible (in principle, edible) MOF based on a γ-cyclodextrin potassium salt, in olive oil (ESI 12, Fig. S24†). The CD-MOF-1 and olive oil were simply stirred together magnetically for 120 minutes to form a homogeneous dispersion that was found to be stable to sedimentation for at least one day. This dispersion exhibited double the CO_2_ uptake (0.18 mmol g^−1^) of olive oil alone (0.09 mmol g^−1^) (ESI 12†). The amount taken up was as predicted based on the uptakes of the olive oil and CD-MOF alone, indicating that, as hoped, the liquid did not penetrate into the pores of the MOF. The fact that this T3PL composition is also biocompatible may open the possibility of biomedical applications such as drug delivery for porous liquids.^23^

## Conclusions

In conclusion, we note the following points: (1) a broad range of porous solids including archetypal MOFs, zeolites and porous organic polymers can be readily dispersed into electrically neutral, chemically and thermally tolerant liquid phases including silicone oils (PDMS), naturally occurring triglyceride oils and organic polymers such as polyethylene glycol derivatives, by simple prolonged magnetic stirring or by brief ball milling; (2) dispersion stability can be high even with as-prepared porous solids, but can also be optimised if required by tuning physical parameters such as the surface–liquid interaction between solid and liquid phases *via* modifying particle size and/or surface of solid or functional group of liquid media, density and viscosity of liquid media; (3) many examples exhibit permanent porosity as evidenced by high gas uptakes, supported by PALS measurements; (4) permanent porosity results from steric hindrance preventing ingress of the liquid into the pores of the solid; (5) examples have been tailored toward natural gas sweetening, ethane/ethene separation and applications that require biocompatibility; (6) taken together, these factors point to T3PLs as a broad platform of tailorable, fluid sorptive materials with wide potential applications.

## Conflicts of interest

There are no conflicts to declare.

## Supplementary Material

SC-011-C9SC05770F-s001
