# Supplementary material for: Type 3 porous liquids based on non-ionic liquid phases – a broad and tailorable platform of selective, fluid gas sorbents
Source: Chem Sci. 2020 Jan 9;11(8):2077–84. doi: 10.1039/c9sc05770f (PMC8150117; doi:10.1039/c9sc05770f)
Supplement: SC-011-C9SC05770F-s001 [file SC-011-C9SC05770F-s001.pdf]

Electronic Supplementary Material (ESI) for Chemical Science.
This journal is © The Royal Society of Chemistry 2020

Type 3 Porous Liquids based on Non-ionic Liquid Phases - a Broad and Tailorable Platform of Selective, Fluid Sorbents

John Cahir,[a] Min Ying Tsang,[a] Beibei Lai,[a] David Hughes,[b] M. Ashraf Alam,[b] Johan Jacquemin,[a] David Rooney,[a] and Stuart L. James[a]

Electronic Supporting Information

Table of Contents

S.I. 1	Materials and measurements…………………………………………..	P.2
S.I. 2	Synthesis of porous solids...	P.4
S.I. 3	Synthesis of porous liquids	P.8
S.I. 4	Characterization (PXRD, IR and TGA)	P.10
S.I. 5	Dispersion stability study	P.15
S.I. 6	Low pressure gas solubility measurements	P.22
S.I. 7	CO_2 uptake kinetic data of selected T3PLs………………………………	P.31
S.I. 8	PALS measurements……………………………………………………	P.32
S.I. 9	Regeneration	P.37
S.I. 10	High pressure gas solubility measurements	P.40
S.I. 11	Ethylene/ethane uptakes……………………………………………….......	P.41
S.I. 12	Biocompatible CD-MOF-1/Olive oil	P.42
	References	P.43

S.I. 1 Materials and measurements

The MOFs and PAF-1 were synthesized according to literature. Zeolites (including Zeolite Sigma – a zeolite branded by Sigma Aldrich, product no. 96096) were purchased from Sigma Aldrich in > 98% purity. All silicone oils, triglyceride oils, halogenated oils and polyethylene glycol derivatives were obtained from Sigma Aldrich UK in >98% purity or Alfa Aesar in >95% purity and were used as obtained without further purification. Genosorb® 1753 was a gift from Clariant International Ltd. PXRD measurements were carried out on a PANanalytical X'Pert Pro X-ray diffractometer. Copper was used as the X-ray source with a wavelength of 1.5405 Å. All experiments were carried out ex-situ using a spinning stage. Diffractograms were typically obtained from 5–50° with a step size of 0.0167°. Infrared spectra were obtained with aPerkin Elmer Spectrum 1. Thermogravimetric Analysis (TGA) were measured by the Analytical Service department of the School of Chemistry and Chemical Engineering (ASEP) using a Mettler Toledo DSC/TGA 1 Star instrument.

Table S1a: Viscosities and boiling points of non-triglyceride oils used in this study.

	Silicone oil 20cst	Silicone oil 50cst	Silicone oil 350cst	Silicone oil 1000cst	Silicone based oil AR20	Fomblin Y oil	Krytox oil	Paraffin oil
Viscosity	20 cst	50 cst	350 cst	1000 cst	20 cst	60 cst	177 cst	172 cst
Boiling point	>140°C	>140°C	>150°C	>200°C	>150°C	c.a. 270°C	c.a. 270°C	370°C
Vapour pressure	< 5 mmHg (25 °C)	< 5 mmHg (25 °C)	< 5 mmHg (25 °C)	< 5 mmHg (25 °C)	< 5 mmHg (25 °C)	-	10-13 mmHg (25 °C)	$1x10^{-3}$ mmHg (25 °C)

Table S1a continued.

	Genosorb® 1753	Polypropylene glycol	Poly(ethylene glycol) PEG-200	Polyethylene bis(2-ethylhexanoate)	Polyethylene glycol dibenzoate	Polyethylene dimethyl ether acrylate
Viscosity	8 cst	80 cst	60 cst	50 cst	110 cst	42 cst
Boiling point	>140°C	c.a. 190°C	c.a. 250°C	c.a. 230°C	c.a. 225°C	c.a. 200°C
Vapour pressure	< 2 mmHg (25 °C)	< 0.08 mmHg (25 °C)	< 0.01 mmHg (25 °C)	< 0.01 mmHg (25 °C)	< 0.01 mmHg (25 °C)	< 0.01 mmHg (25 °C)

Table S2 Viscosities, smoke points and degrees of unsaturation of triglycerides oils used in this study

	Olive oil	Castor oil	Sesame oil	Sunflower oil	Safflower oil	Soy bean oil	Corn oil
Viscosity	79.1	915	52	29.3	33.5	30.1	32.8
Smoke point	c.a. 200°C	c.a.310°C	c.a. 350°C	c.a. 230°C	c.a. 250°C	c.a. 300°C	c.a. 160°C
**Unsaturation (Mono: Poly) vs saturation in side chains**	(78:8):14	(5:89):7	(41:44):15	(20:69):11	(14:79):7	(25:60):15	(25:62):13
**Vapour pressure**	< 1x10^{-3} mmHg (25 ^{o}C)	< 1x10^{-3} mmHg (25 ^{o}C)	< 1x10^{-3} mmHg (25 ^{o}C)	< 1x10^{-3} mmHg (25 ^{o}C)	< 1x10^{-3} mmHg (25 ^{o}C)	< 1x10^{-3} mmHg (25 ^{o}C)	< 1x10^{-3} mmHg (25 ^{o}C)

S.I. 2 Synthesis of porous solids

Synthesis of ZIF-8[1]: *Solution synthesis*: $Zn(NO_3)_2.6H_2O$ (1.00g, 3.36 mmol) was dissolved in MeOH (20 mL) in a conical flask. 2-methylimidazole (1.10g, 13.44 mmol) was dissolved in MeOH (20 mL) and added to the former solution. The mixture was stirred overnight at room temperature. The precipitate was collected by filtration, washed with MeOH (3x 20 mL) and dried in air. Activation conditions: 3 hours at 200 ^{o}C. *Ball mill synthesis*: $Zn_5(CO_3)_2(OH)_6$ (0.175g, 0.32 mmol) and 2-methylimidazole (0.393g, 4.79 mmol) were added to a 25 mL ball mill jar with a 13.6 g ball bearing, followed by the addition of 50µL MeOH. The mixture was milled for 30 min at 20Hz. The obtained solid was washed with EtOH (3 x 10 mL). Activation conditions: 3 hours at 150 ^{o}C.

Synthesis of HKUST-1[2]: *Solution synthesis*: $Cu(NO_3)_2.2.5H_2O$ (0.5g, 2.15 mmol) was dissolved in MeOH (10 mL) in a conical flask. Benzene-1,3,5-tricarboxylic acid (0.91g, 4.30 mmol) was dissolved in a MeOH (10 mL) and mixed with the former solution. The mixture was stirred overnight at room temperature. The blue precipitate was collected by filtration,washed with MeOH (3 x 10mL) and dried in air. Activation conditions: 5 hours at 200°C. *Ball mill synthesis*: $Cu(OH)_2$ (0.21g, 2.15 mmol) and benzene-1,3,5-tricarboxylic acid (0.29g, 1.37 mmol) were added to a 25 mL steel ball mill jar with a steel 13.6 g ball bearing, followed by MeOH (0.5 mL). The mixture was milled for 15 min at 25Hz. The obtained solid was washed with EtOH (3 x 20 mL) and dried in air. Activation conditions: 3 hours at 200°C.

Synthesis of Al(fumarate)(OH)[3]: $Al_2(SO4)_3.18H_2O$ (1.225 g, 3.58 mmol) was dissolved in 15 mL deionised water in a conical flask. Fumaric acid (415 mg, 3.57 mmol) and NaOH (286.4 mg, 7.16 mmol) were dissolved in deionised water (15 mL) and mixed with the former solution. The mixture was heated at 60°C for 3 hours and cooled to room temperature. The off-white precipitate was collected by centrifugation and washed with deionised water (2 x 15 mL) and MeOH (2 x 15 mL). The obtained solid was dried in air. Activation conditions: 3 hours at 200°C.

Synthesis of SIFSIX-3-Zn[4]: $ZnSiF_6$ (100 mg, 0.48 mmol) was dissolved in MeOH (3 mL) in a vial. Pyrazine (77.2 mg, 0.96 mmol) was dissolved in MeOH (2 mL) and mixed with the former solution. The mixture was stirred overnight at room temperature in air. A pale yellow precipitate was collected by centrifugation, washed with MeOH (2 x 5 mL), dried under vacuum and stored under N_2. Activation conditions: 3 hours at 55°C under vacuum.

Synthesis of SIFSIX-3-Cu[5]: $CuSiF_6$ (98.7 mg, 0.48 mmol) was dissolved in MeOH (3 mL) in a vial. Pyrazine (77.2 mg, 0.96 mmol) was dissolved in MeOH (2 mL) and mixed with the former solution. The mixture was stirred overnight at room temperature under air. A pale yellow precipitate was collected by centrifugation, washed with MeOH (2 x 5 mL), dried under vacuum and stored under N_2. Activation conditions: 3 hours for 55^oC under vacuum.

Synthesis of UiO-66[6]: $ZrCl_4$ (1.29 g, 5.54 mmol) was dissolved in DMF (30 mL) in a conical flask. Terephthalic acid (0.9 g, 7.76 mmol) was dissolved in DMF (30 mL) and mixed with the former solution. The mixture was transferred to an autoclave,heated at 120°C overnight and allowed to cool to room temperature. The off-white precipitate was collected by centrifugation, washed with DMF (2 x 20 mL) andMeOH (20 mL). The obtained solid was dried overnight in air. Activation condition: 2 hours at 200^oC under vacuum.

Synthesis of UiO-66-NH_2[7]: $ZrCl_4$ (200 mg, 0.86 mmol) was dissolved in a mixture of DMF (5 mL) and H_2O (0.1 mL) in a conical flask. 2-amino-terephthalic acid (155.5 mg, 0.86 mmol) was dissolved in DMF (5 mL) and mixed with the former solution. The mixture was transferred to an autoclave and heated at 120°C overnight and allowed to cool to room temperature. The pale yellow precipitate was collected by centrifugation, washed with DMF (2 x 10 mL) and MeOH (10 mL), and dried overnight in air. Activation condition: 2 hours at 200^oC under vacuum.

Synthesis of ZIF-67[8]: $Co(NO_3)_2.6H_2O$ (1.50g, 5.15 mmol) was dissolved in deionised water (50 mL) in a conical flask. 2-methylimidazole (1.69g, 20.58 mmol) and triethylamine (2mL) were dissolved in deionised water (50 mL) and mixed with the former solution. The mixture was stirred overnight at room temperature under air. The purple precipitate was collected by filtration and washed with deionised water (3 x 20 mL) and dried in air. Activation conditions: 3 hours at 150^oC.

Synthesis of ZIF-90[9]: $Zn(NO_3)_2.6H_2O$ (0.51 g, 1.72 mmol) was dissolved in DMF (10 mL) in a conical flask. 2-imidazolecarboxaldehyde (0.79g, 8.27 mmol) and dihexylamine (1.2 mL) were dissolved in DMF (10 mL) and mixed with the former solution. The mixture was stirred overnight at room temperature under air. The white precipitate was collected by centrifugation and washed with DMF (2 x 20 mL) and MeOH (2 x 20 mL). Activation conditions: 2 hours at 150^oC.

Synthesis of MOF-801[10]: $ZrCl_4$ (2.33 g, 9.99 mmol) was dissolved in deionised water (25 mL) in a conical flask. Fumaric acid (1.16g, 9.99 mmol) was dissolved in acetic acid (25 mL) and mixed with the former solution. The mixture was heated to 95°C for 1 hour under air. The off-white precipitate was collected by centrifugation, washed with deionised water (3 x 50 mL) and dried in air. Activation conditions: 2 hours at 200°C.

Synthesis of MIL-53(Al)[11]: $Al(NO_3)_3.H_2O$ (1.00g, 2.67 mmol) was dissolved in deionised water (25 mL) in a conical flask. Terephthalic acid (0.996 g, 5.99 mmol) was dissolved in DMF (25 mL) and mixed with the former solution. The mixture was transferred to an autoclave and heated to 150°C for 3 days. The white precipitate was collected by centrifugation and washed with DMF (10 mL) and deionised water (3 x 10 mL). Activation conditions: 2 hours at 200°C.

Synthesis of CAU-10-H[12]: Aluminium sulfate hydrate ($Al_2(SO_4)_3 \cdot 18H_2O$) (5.05 g, 7.5 mmol) was dissolved in deionized water (25 mL), and isophthalic acid (1.32 g, 7.9 mmol) was dissolved in DMF (7 mL). The two solutions were combined in a round bottom flask. The combined mixture was heated under reflux for 117 hrs. After cooling the precipitate was collected by filtration and redispersed in deionized water (200 mL) by stirring. The solid was collected by filtration and dried for 2 days at 100 °C. Activation conditions: 5 days at 120°C under vacuum.

Synthesis of CD-MOF-1[13]: Gamma cyclodextrin (1.30 g, 1 mmol) and potassium hydroxide (0.45 g, 8 mmol) were dissolved in deionized water (20 mL). The aqueous solution was filtered and MeOH vapour was allowed to diffuse into the solution over one week. Colourless cubic crystals (1.20 g) were collected by filtration, washed with MeOH (2 x 30 mL) and collected by centrifugation. Activated conditions: for 24 hours at 50°C under vacuum after solvent exchange with dichloromethane for 3 days.

Synthesis of OPOSS decorated HKUST-1[14]: OPOSS was synthesized according to known literature methods where 3-aminotrimethoxysilane (0.28 mL, 1.1 mmol) was added to isooctyl trimethoxysilane (3 mL, 7.7 mmol) in an autoclave with 25 wt.% ammonia solution (13.68 mL) and heated at 150 ^{o}C for 24 hours. The resulting viscous liquid was partitioned using a 50:50 mixture of water and dichloromethane. The dichloromethane layer was separated, washed three times with water (40 mL) and dried over sodium sulfate. The solvent was removed under reduced pressure (400 mbar) with a bath temperature of 50 ^{o}C. OPOSS was decorated

onto the surface of HKUST-1 according to literature methods where HKUST-1 (1.0 g, 0.02 mol) was added to OPOSS (0.3 g 1.2 mmol) in hexane solution (60 mL) and allowed to reflux under nitrogen for 24 hours. The remaining solid was filtered and dried for 24 hours at 100°C.

Synthesis of PAF-1[15]: *Tetrakis(4-bromophenyl)methane*: To a three-necked round-bottom flask containing bromine (6.4 mL, 19.9 g), tetraphenylmethane (2.0 g, 6.24 mmol) was added stepwise in small portions under vigorous stirring at room temperature (25 °C). The resulting solution was stirred for 60 min and cooled to 0 °C. Ethanol (25 mL) was added slowly, and the reaction mixture allowed to warm to room temperature overnight. The precipitate was collected by filtration and washed with saturated aqueous sodium hydrogensulfite solution (25 mL) and water (100 mL). After drying at 80 °C for 24 h under vacuum, tetrakis(4-bromophenyl) methane was recrystallized from EtOH/CH_2Cl_2 to afford a yellow solid. *PAF-1*: Tetrakis(4-bromophenyl)methane (509 mg, 0.8 mmol) was added to a solution of 2,2'-bipyridyl (565 mg, 3.65 mmol), bis(1,5-cyclooctadiene)nickel(0) (1.0 g, 3.65 mmol), and 1,5-cyclooctadiene (0.45 mL, 3.65 mmol) in anhydrous DMF/THF (60 mL/90 mL). The mixture was stirred overnight at room temperature under nitrogen atmosphere. HCl (6 m, 60 mL) was added slowly, and the resulting mixture stirred for 12 h. The precipitate was collected by filtration, washed with methanol and water, and dried at 150 °C for 24 h under vacuum (80 mbar) to give PAF-1 as a white powder. Elemental analysis: Theoretical: C: 93.71%, H: 6.29%; experimental: 93.88%, H: 6.12%.

Synthesis of silver zeolite AgA [16]: Silver nitrate (8.49g, 50 mmol) was dissolved in distilled water (100 mL). Zeolite 5A (1.0 g) was added and the mixture stirred for 5 hours at 80 °C. The solid was filtered and washed with distilled water (3× 30 mL). The remaining solid was filtered and dried in air at room temperature.

S.I. 3 Synthesis of porous liquids

Two general methods were used:

Method 1: Activated porous solid powder (typically 180 - 200 mg, up to 25 wt %) was mixed with the chosen liquid (typically 1.3 - 1.5 ml) by vigorous magnetic stirring for 1-2 hours until formation of homogeneous suspension.

Method 2: Unactivated solid powder (180 - 200 mg) was mixed with the chosen liquid (1.3 - 1.5 ml) by vigorous stirring for 1-2 hours until a formation of homogeneous suspension. The suspension was heated under reduced pressure as appropriate to activate of the porous solid component.

In the current study, porous liquids were primarily obtained by *Method 1*. Selected examples are given below:

Zeolite 5A/castor oil (12.5wt%): Activated zeolite 5A powder (180 mg) was added to castor oil (1.3 mL, density: 0.93 gmL^{-1}) and stirred at 600 rpm with a magnetic stirrer in a 10mL glass vial. The dispersion was characterized by powder X-ray diffraction (PXRD) and IR spectroscopy (**Figures S1** and **S2**).

PAF-1/Genosorb® (12.5wt%): Activated PAF-1 powder (180 mg) was added to castor oil (1.3 mL) and stirred at 600 rpm with a magnetic stirrer in a 10mL glass vial. The dispersion was characterized by CHNS analysis and IR spectroscopy (**Figure S2).**

ZIF-8/PDMS (12.5wt%): Activated ZIF-8 powder (180 mg) was added to PDMS (1.3 mL) in a 10mL glass vial and stirred at 600 rpm with a magnetic stirrer. The dispersion was characterized by powder X-ray diffraction (PXRD) and IR spectroscopy and (**Figures S1** and **S2**).

Al(fum)(OH)/PDMS (12.5wt%): Activated Al(fumarate)(OH) powder (180 mg) was added to PDMS (1.3 mL) in a 10mL glass vial and stirred at 600 rpm with a magnetic stirrer. The dispersion was characterized by powder X-Ray Diffraction (PXRD) and IR spectroscopy (**Figures S1** and**S2).**

SIFSIX-3-Zn/Fomblin® Y (6.3wt%): Activated SIFSIX-3-Zn powder (180 mg) was added to Fomblin® Y (0.7 mL, density: 1.89 gmL^{-1}) in a 10 mL glass vial and stirred at 600 rpm with a

magnetic stirrer. The dispersion was characterized by powder X-Ray Diffraction (PXRD) and IR spectroscopy **Figures S1** and **S2**.

HKUST-1/Olive oil (12.5wt%): Activated HKUST-1 powder (180 mg) was added to olive oil (1.3 mL, density: 0.93 gmL^{-1}) in a 10mL glass vial and stirred at 600 rpm with a magnetic stirrer. The dispersion was characterized by powder X-Ray Diffraction (PXRD) and IR spectroscopy (**Figures S1** and **S2).**

S.I. 4 Characterization of porous liquids

All porous liquids were characterized by Powder X-Ray Diffractometry (PXRD) and IR spectroscopy (**Figures S1** and **S2**). All PXRD patterns show peaks corresponding to the crystalline solid component, indicating that the solid components remain intact and crystalline, superimposed on broad features due to the liquid components.

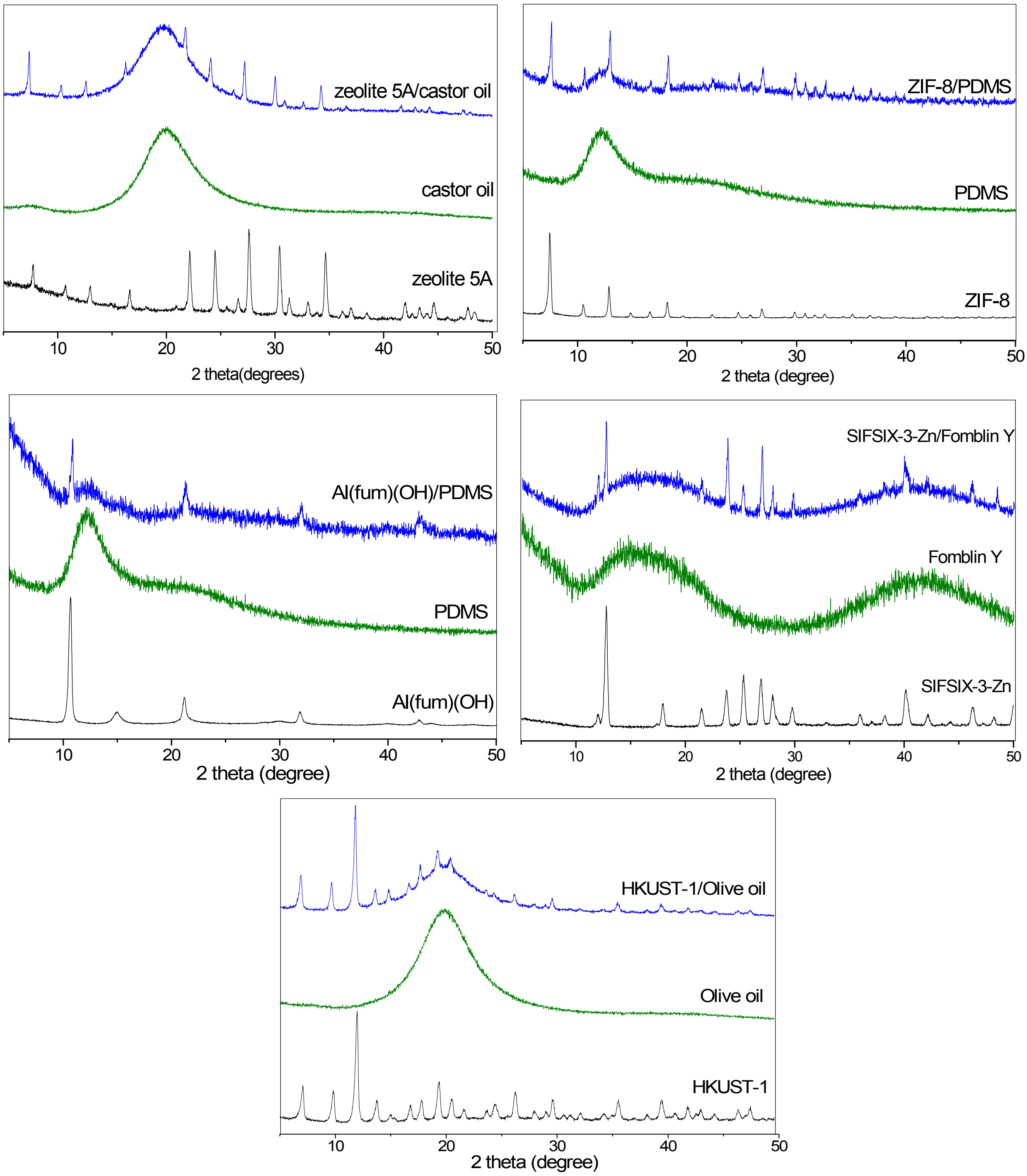

Figure S1: PXRD patterns of selected porous liquids: zeolite 5A/castor oil, ZIF-8/PDMS, Al(fum)(OH)/PDMS, SIFSIX-Zn/Fomblin Y, HKUST-1/olive oil.

IR spectra show a combination of the features expected from the solid and liquid components.

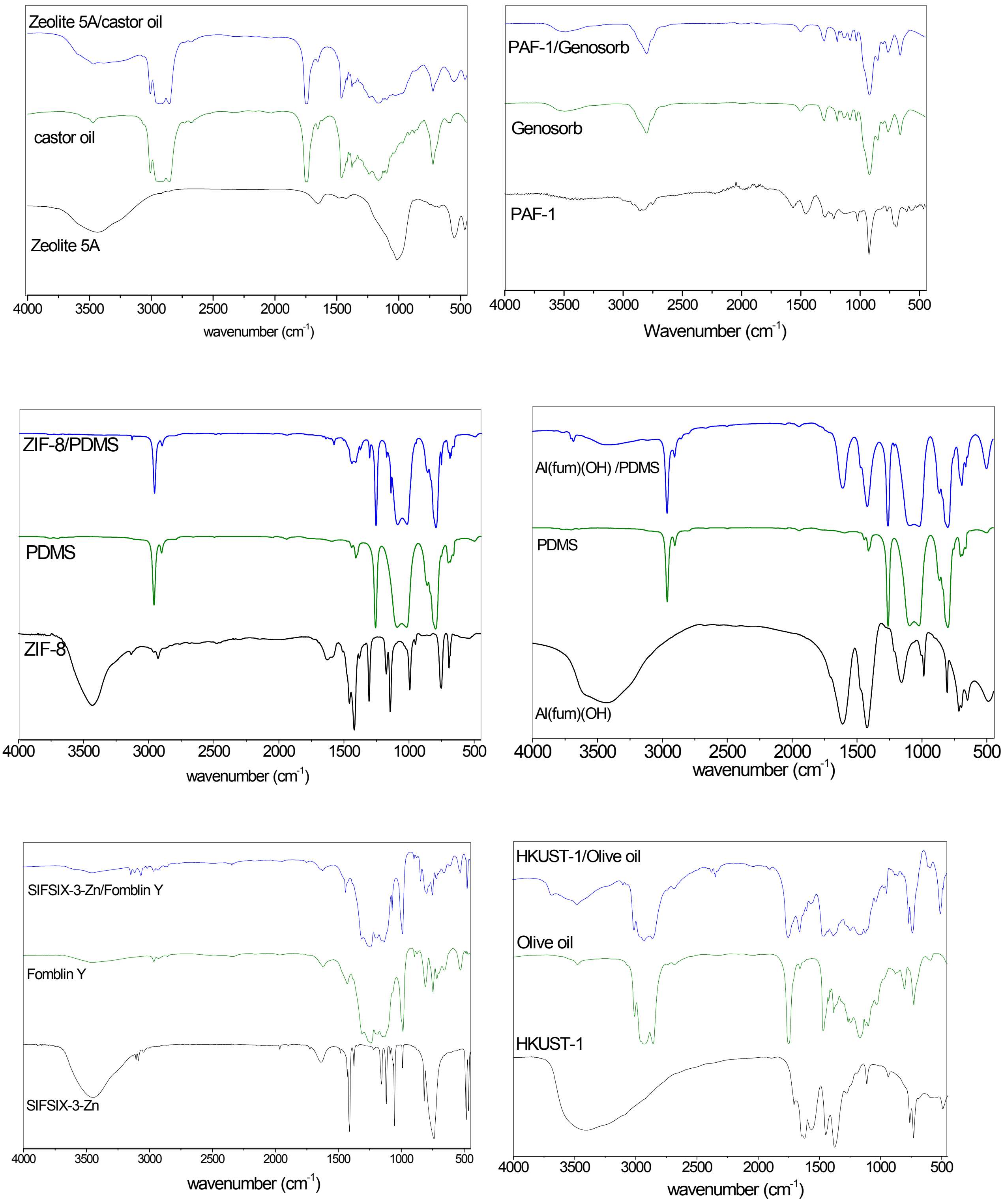

Figure S2: IR spectra of selected porous liquids: zeolite 5A/castor oil, PAF-1/Genosorb®, ZIF-8/PDMS, Al(fum)(OH)/PDMS, SIFSIX-Zn/Fomblin Y, HKUST-1/olive oil.

Thermogravimetric analysis (TGA) of selected examples (**Figures S3-6**) confirmed that the thermal stability was as expected based on the thermal stabilities of the individual solid and liquid components. It is notable that in some cases, especially with silicone polymers, thermal stability could be high (> 300 ^{o}C).

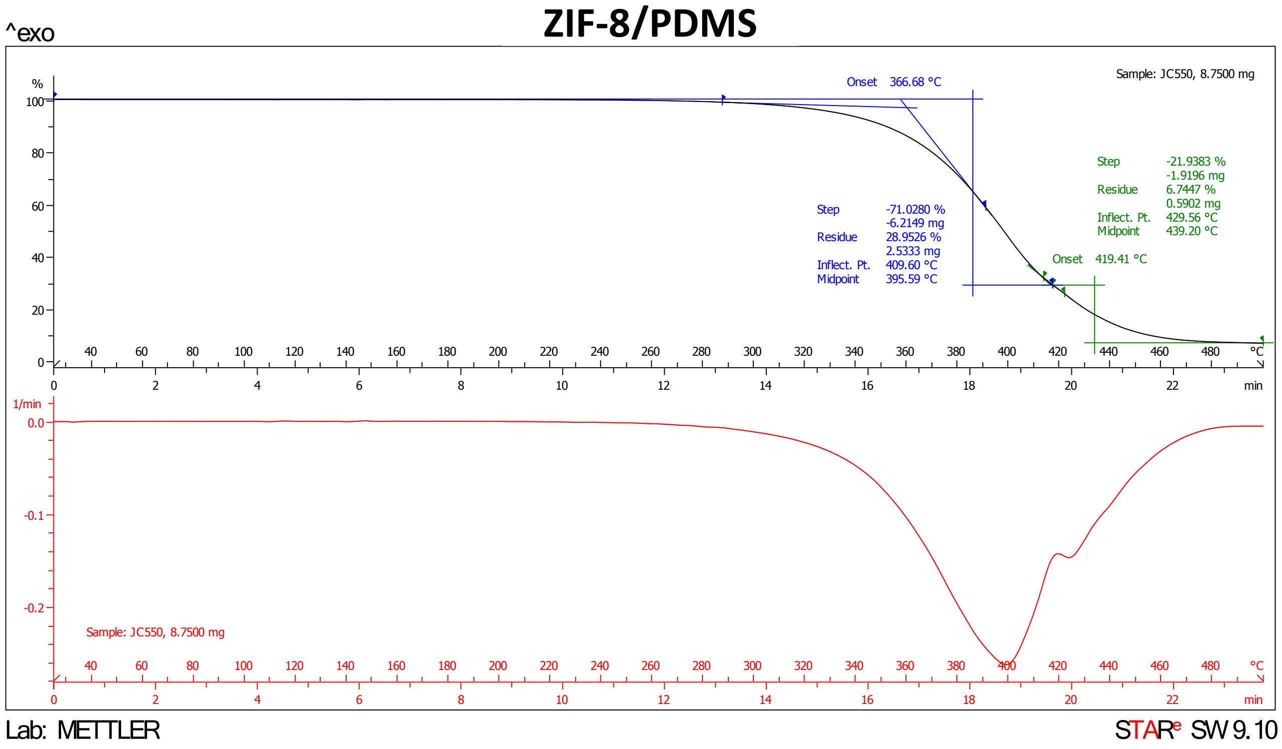

Figure S3: TGA and DSC curves for ZIF-8/PDMS.

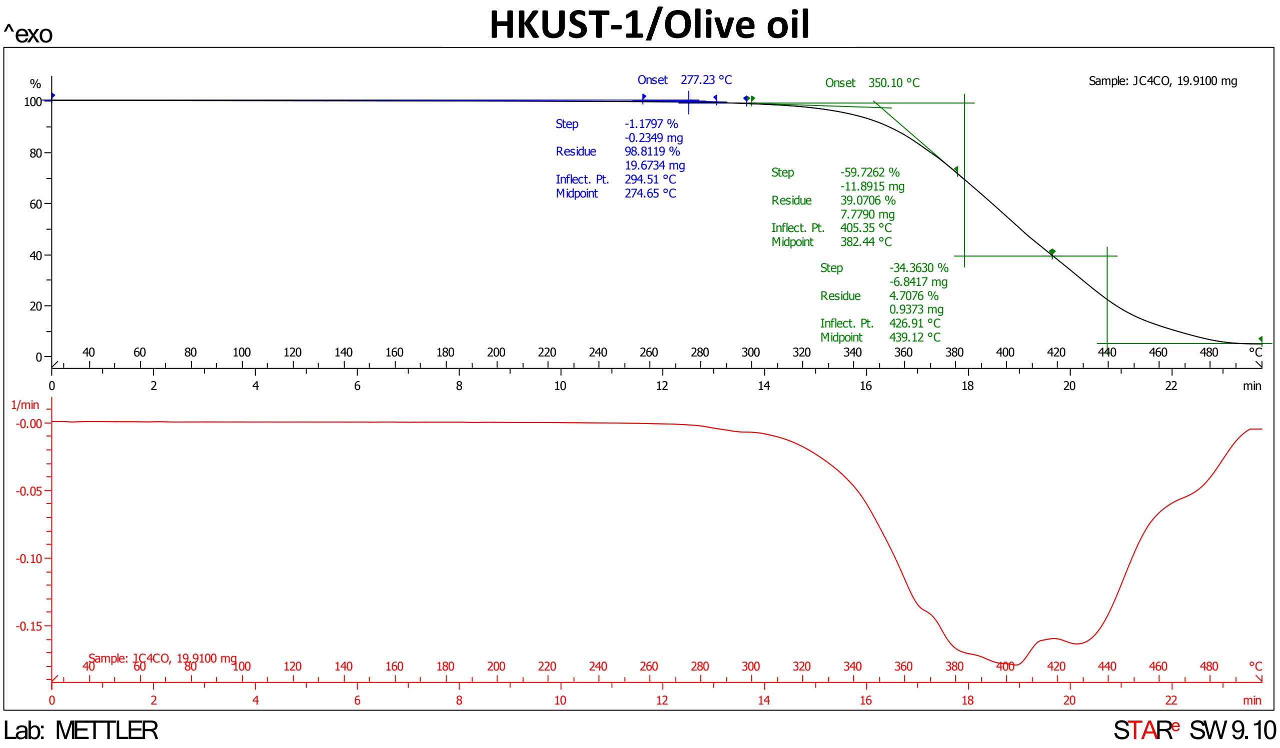

Figure S4: TGA and DSC curves for HKUST-1/Olive oil.

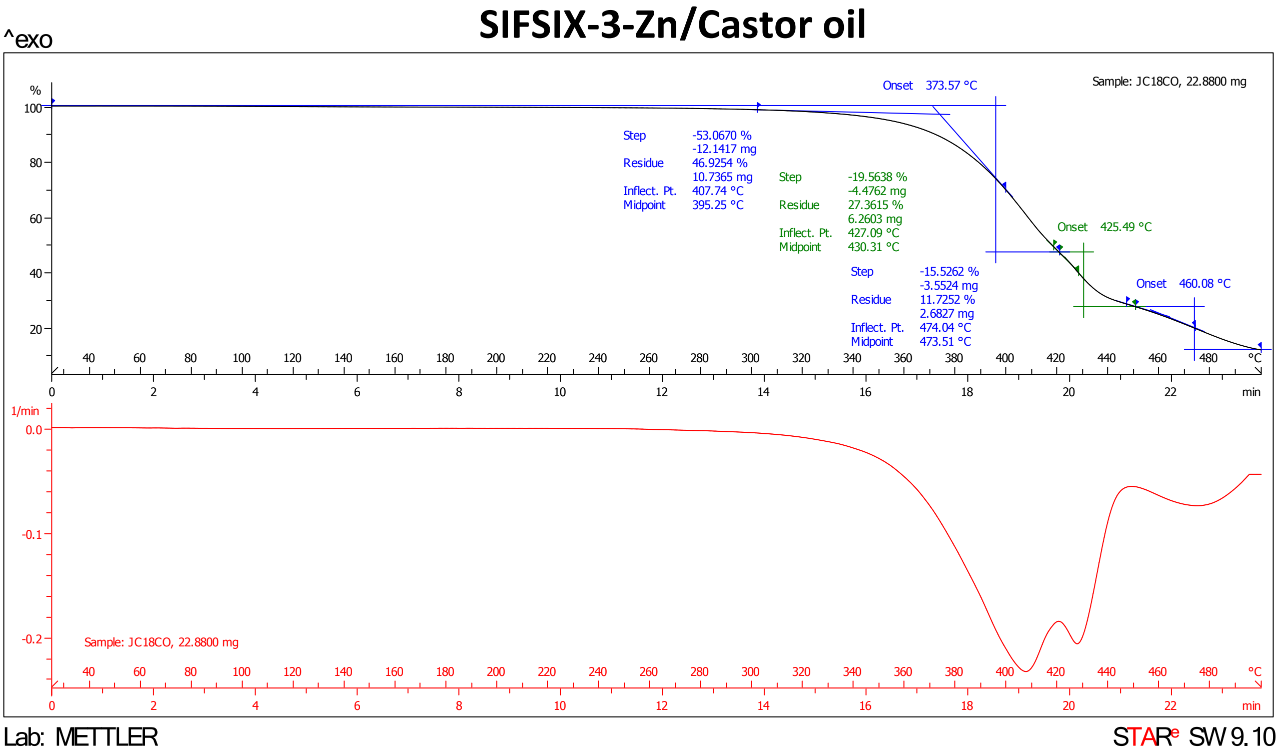

Figure S5: TGA and DSC curves for SIFSIX-3-Zn/castor oil.

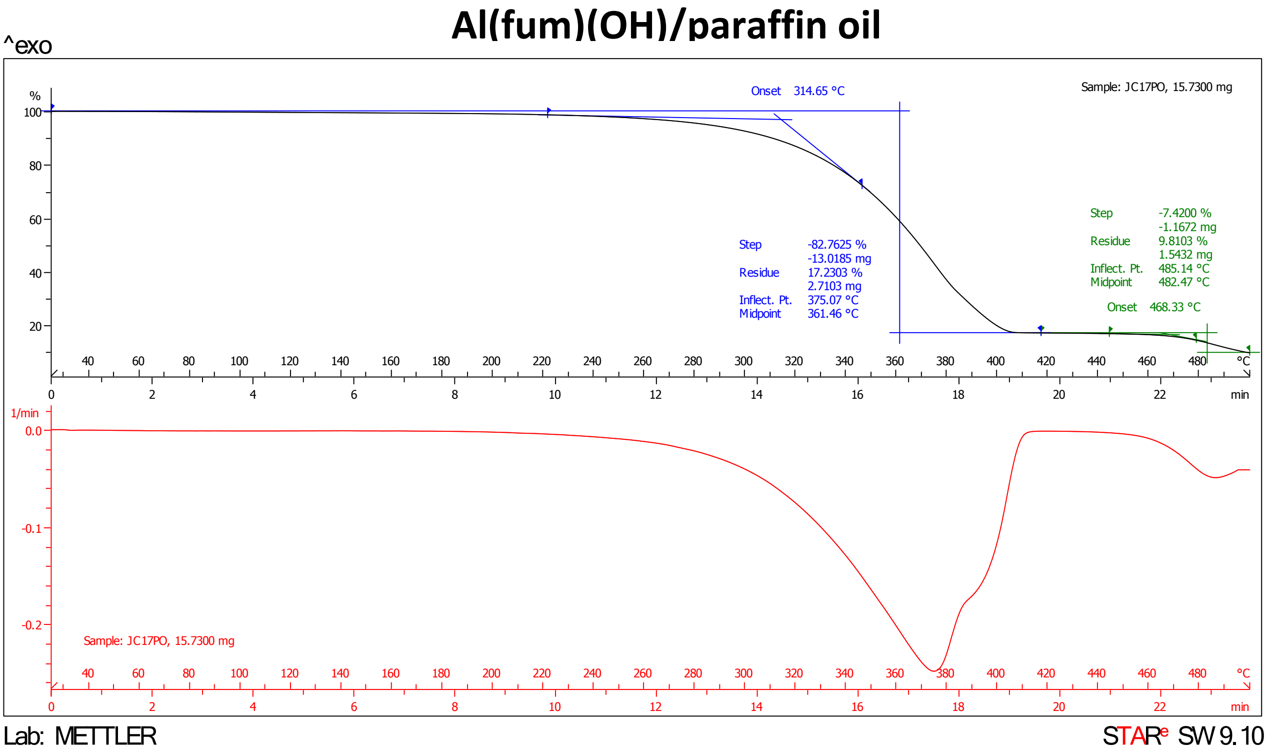

Figure S6: TGA and DSC curves for Al(fum)(OH)/paraffin oil.

S.I. 5 Dispersion stability study

The stability of the dispersions to sedimentation or flotation was screened by visual observation, complimented for selected examples by quantitative analysis through PXRD as shown below. Four methods were used to enhance dispersion stability: i) reducing the particle size; ii) increasing the attractive interaction between solid and liquid phase; iii) increasing the viscosity of liquid phase; iv) matching the densities of solid and liquid phases (**Figures S7 to S14**).

i) *Reducing the particle size*

Dispersion stability is expected to be greater for smaller particles due to the greater surface area for interaction between the particles and liquid. Correspondingly, reducing the particle size of HKUST-1 particle size by brief ball milling from ca. 400 nm to ca. 50nm, the stability of HKUST-1/PDMS dispersions increased from ca. 1 day to ca. 3 days as shown by PXRD analysis of the upper layer (**Figure S7**). Similarly, Al(fum)(OH) was synthesised at three different particle sizes by varying the solution concentration during synthesis (**Figure S8**). This provided Al(fum)(OH) particles with three different size ranges (as shown by PXRD and SEM, **Figure S9**). The dispersion with the largest particles sedimented in less than one day, whilst that with the smallest particles was stable for more than 1 month.

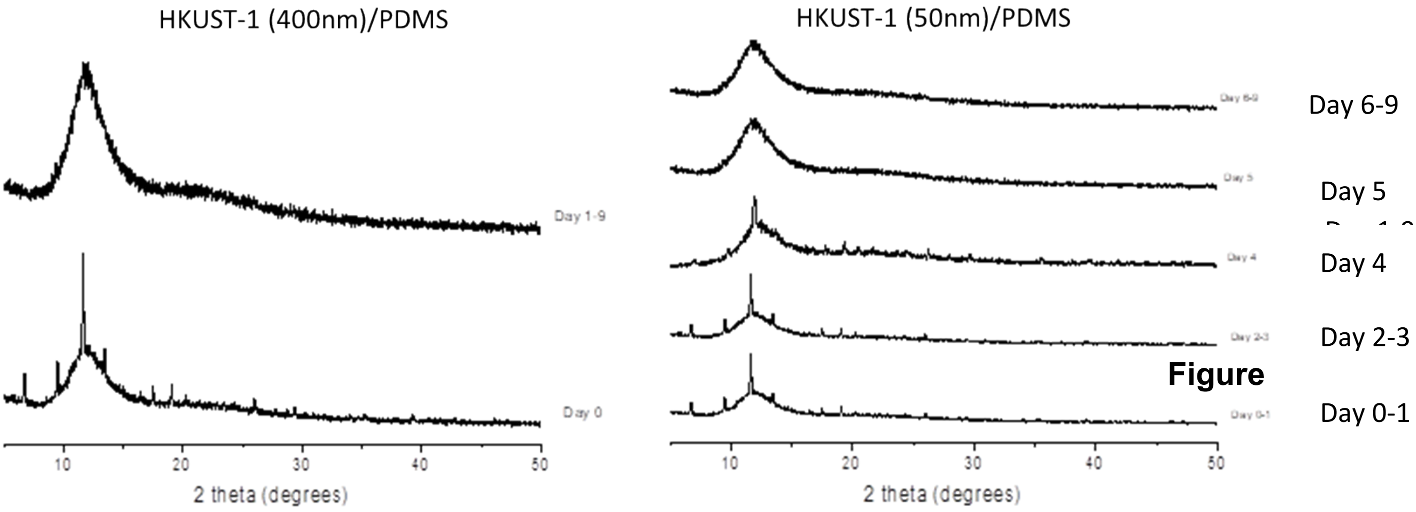

Figure S7: HKUST-1/PDMS dispersion analysis with particle sizes 400nm and 50nm).

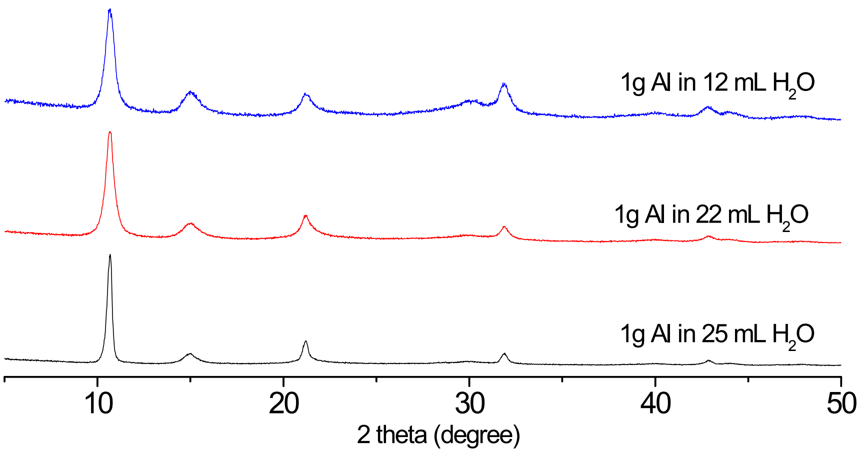

Figure S8: PXRD patterns for Al(fum)(OH) of various particle sizes: 700nm - 1μm (black), 400-600nm (red) and 300-500nm (blue), concentrations for the aluminium precursor during synthesis are indicated for each trace.

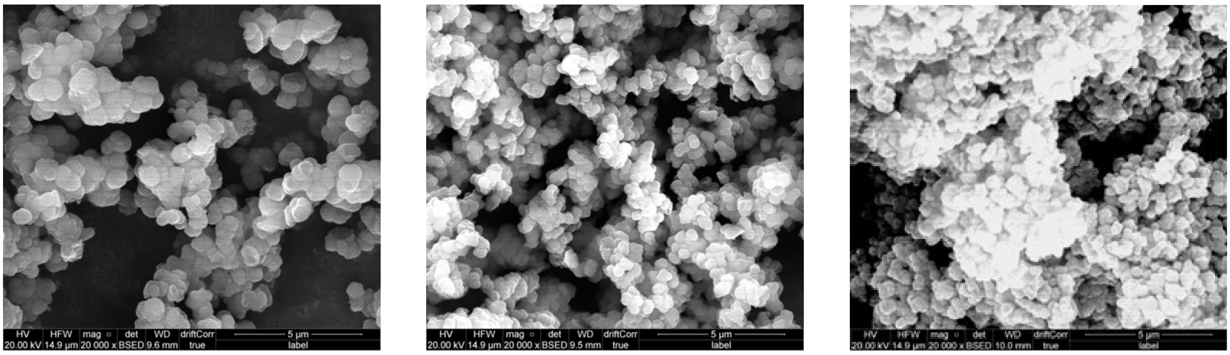

1g Al in 25ml H_2O 700nm – 1μm | 1g Al in 22ml H_2O 400nm – 600nm | 1g Al in 12ml H_2O 300nm – 500nm

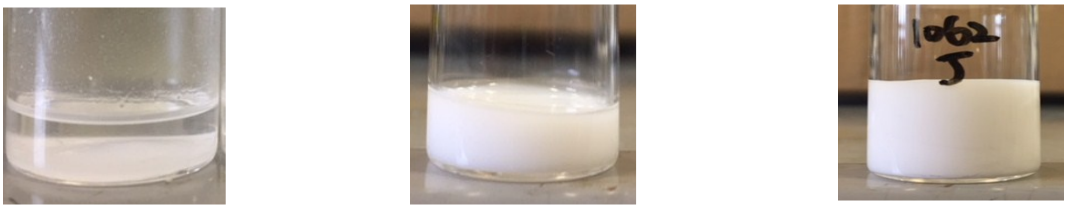

Figure S9: SEM images of Al(fum)OH samples with different particle sizes Photographs of dispersions of Al(fum)(OH) in PDMS with different particle sizes after 1 week, showing greater stability with smaller particle size.

ii) *Increasing the attractive interactions between the MOF and liquid phase*

Replacing half of the methyl groups of PDMS with phenyl groups, as in poly(phenylmethyl)siloxane, PPDMS) was expected to increase the attraction of the liquid to surface of HKUST-1 and was indeed found to give more stable dispersions (**Figure S10**).

A

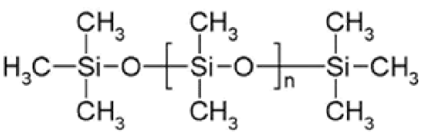

Silicone oil; poly(dimethylsiloxane)

B

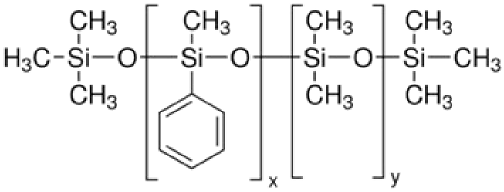

poly(phenylmethylsiloxane)

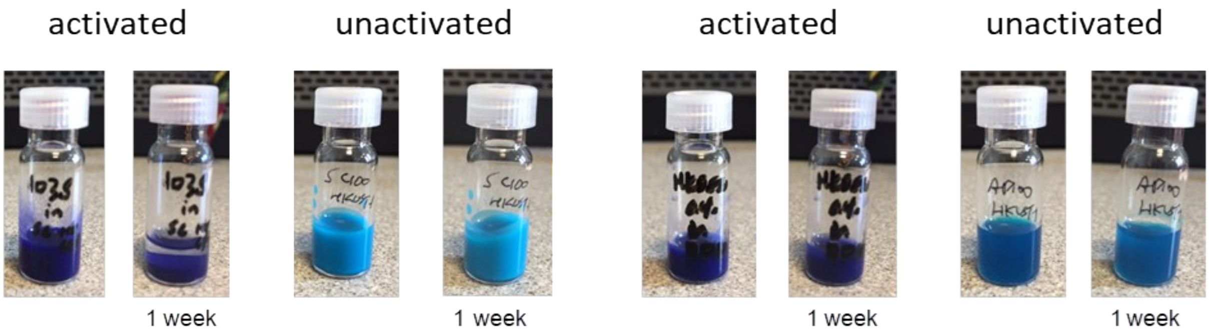

Figure S10: Dispersions of HKUST-1 in (A) PDMS and (B) (PPDMS).

As an alternative approach, functionalising the surface of HKUST-1 particles with OPOSS silsesquioxane cages improved the stability of dispersions in PDMS (**Figure S11**).

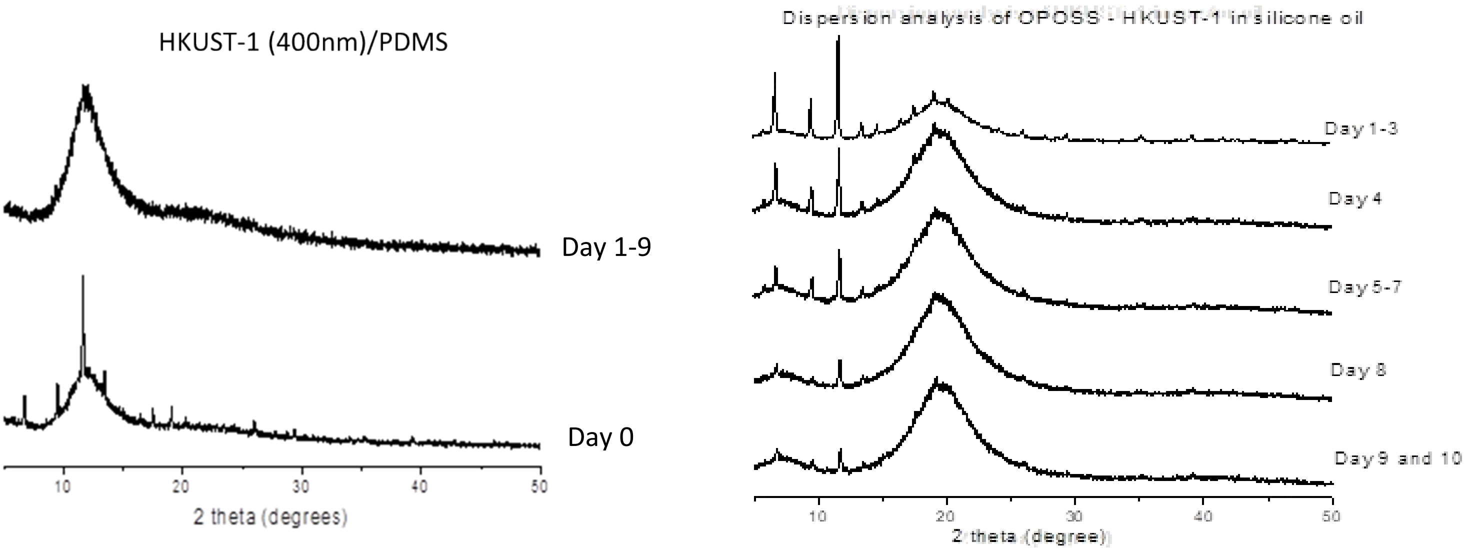

Figure S11a: PXRD monitoring of dispersion showing the greater stability of OPOSS-modified HKUST-1 in silicone oil compared to unmodified HKUST-1 in silicone oil.

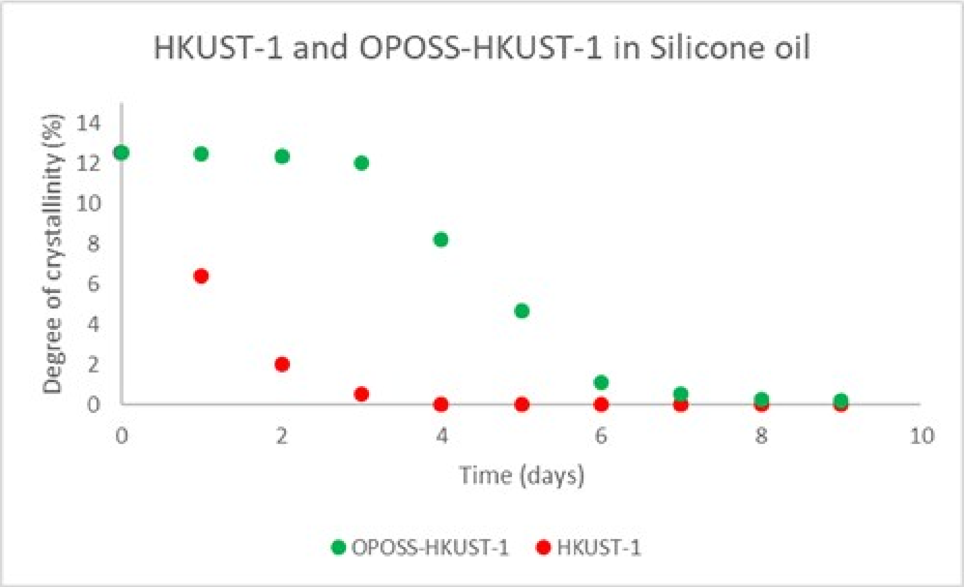

Figure S11b: The degree of crystallinity as a function of time. (Figure S14).

iii) Increasing the viscosity of the liquid phase

Liquid phases with high viscosity liquid phase can retard the sedimentation of solid particles as seen by the different stabilities of HKUST-1 dispersions in PDMS of different viscosities (Figure S12).

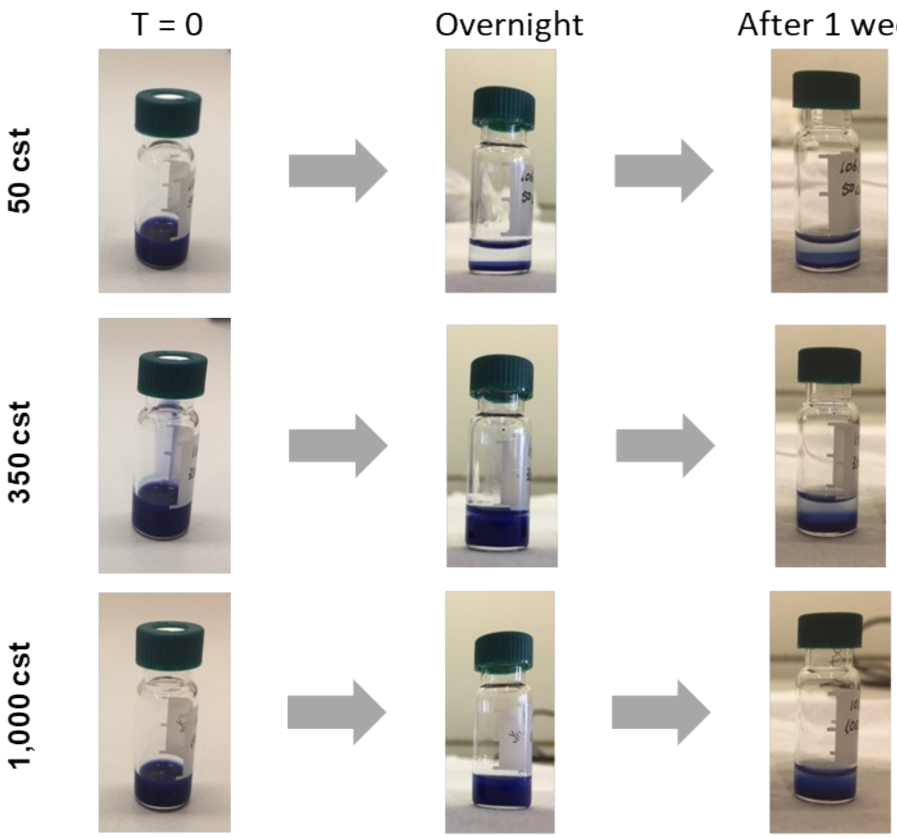

Figure S12: Photographs of dispersions of HKUST-1 in PDMS with different viscosities (50 cst, 350 cst and 1000 cst) showing slower sedimentation in the higher viscosity oils.

iv) Matching the densities of solid and liquid phases

Dispersions of the highly fluorinated MOF SIFSIX-3-Zn in liquids such as silicone oils or triglyceride oils were found to be unstable, settling out within an hour. This can be ascribed to its high crystallographic density of 1.57 g/cm^3 [13]. Use of an oil with high density, specifically the fluorinated oil Fomblin ® Y (density: 1.88 g/cm^3; cf. silicone oil density: 0.96 g/cm^3) was found to give far more stable dispersions.

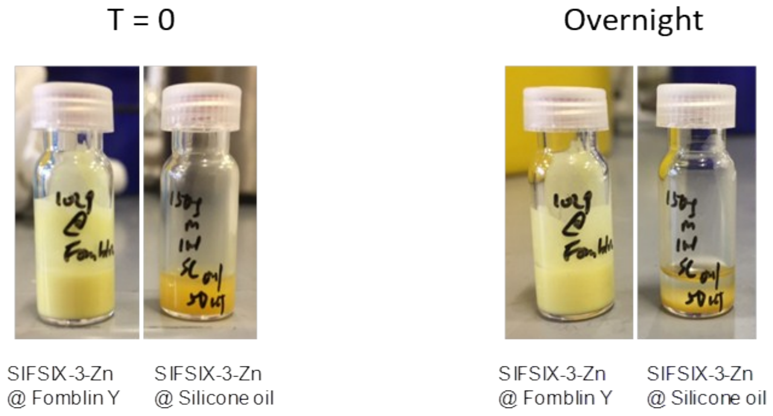

Figure S13: Dispersion of SIFSIX-3-Zn in high density Fomblin Y oil and lower density PDMS.

The dispersion stability was analysed for selected examples by PXRD analysis of the upper layer of the suspension (determined by the penetration of X-Rays) and shown graphically in **Figure S14**. Integrals of the trace for the respective components of the porous liquid were obtained by a variation of Rietveld refinement previously reported.[17] The ratios of the integrals were calculated by simple division and normalised to 12.5% (in relation to the theoretical amount of crystalline material present in the upper layer for a stable dispersion). The calculation was performed each day for nine days and results are plotted graphically in Figure 2b.

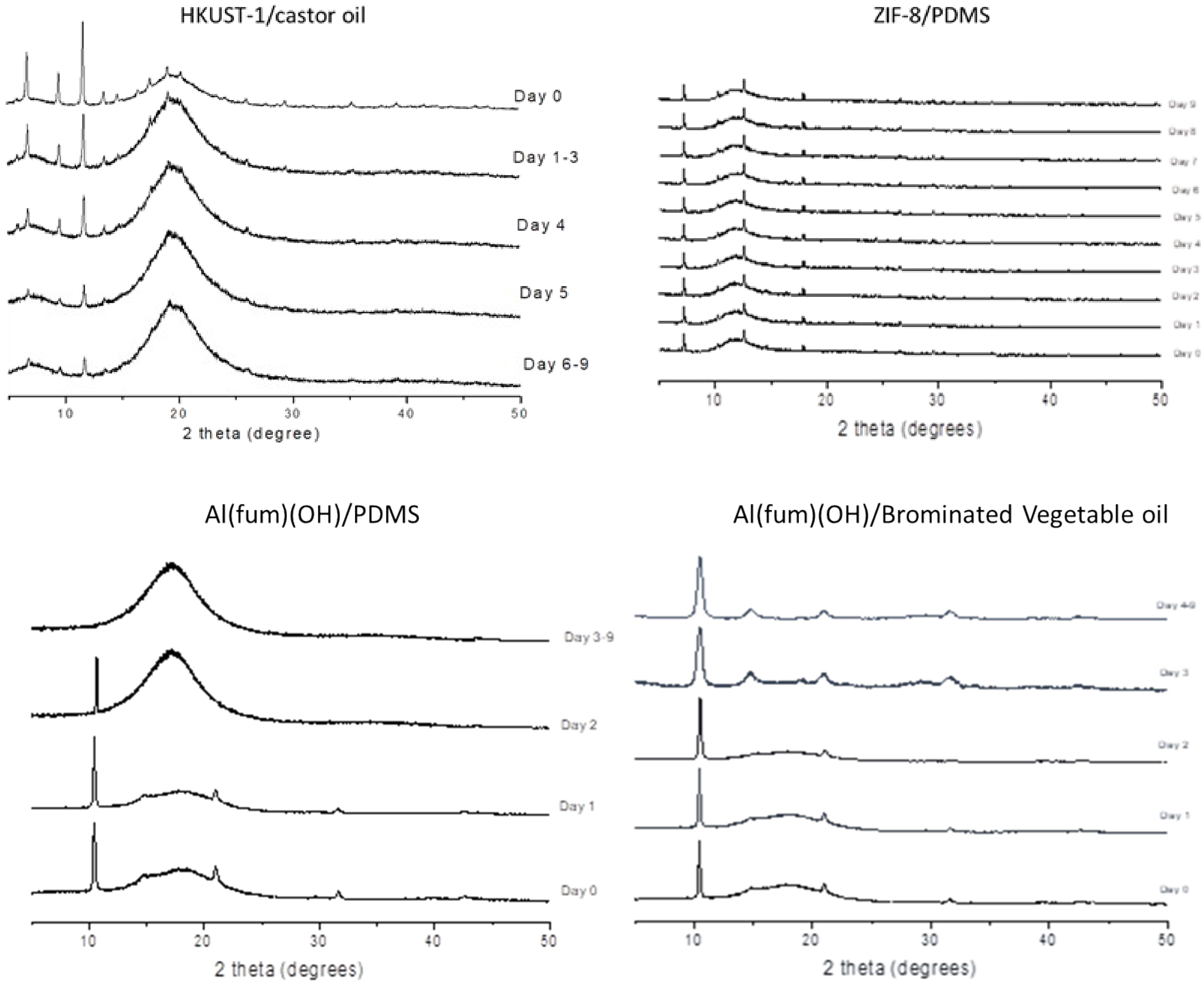

Figure S14: PXRD analysis of stable dispersion of HKUST-1/castor oil and ZIF-8/silicone oil over a period of 10 days.

S.I. 6 Low pressure gas solubility measurements

Gas uptake studies were carried out using an isochoric method described elsewhere[18]. The measurements were carried out at 298K and the average equilibrium pressure was ca. 0.85 bar (**Figure S15**). Total internal volume of the apparatus was calibrated with corresponding gases before measurement. Equilibrium was deemed to be reached when the system pressure was unchanged for at least 2 hours.

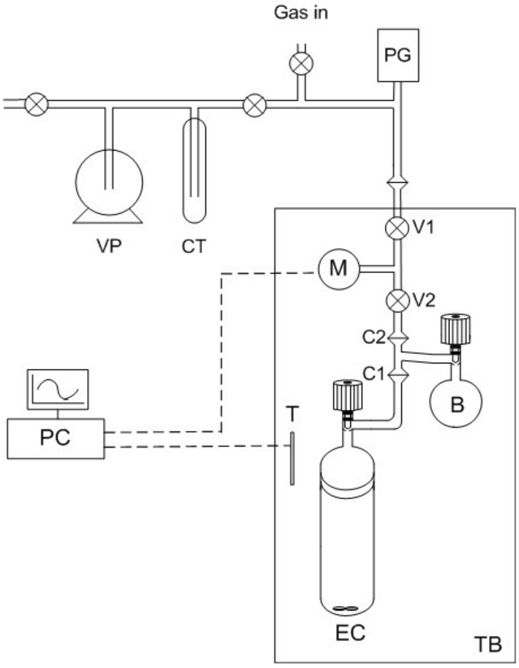

Figure S15: Apparatus for isochoric gas solubility measurement.

Gas uptakes of the pure porous solids were measured to compare with literature values (**Table S3**). Gas uptakes of pure liquid media were also measured for comparison with the gas uptakes of porous liquids. All porous liquids consisted of 12.5 wt% porous solid content, unless otherwise stated.

Table S3: CO_2 uptake of pure porous solids

	Experimental (mg/g (mmol/g))	Literature (mg/g (mmol/g))
**MOFs**		
HKUST-1	170.76 (3.88)	197.61 (4.49)[19]
ZIF-8	36.97 (0.84)	43.13 (0.98)[20]
Al(fum)(OH)	95.59 (2.17)	91.98 (2.09)[21]
SIFSIX-3-Zn	118.83 (2.70)	112.23 (2.55)[22]
SIFSIX-3-Cu	109.14 (2.48)	---
UiO-66	79.22 (1.80)	55.89 (1.27)[23]
UiO-66-NH2	90.66 (2.06)	102.10 (2.32)[24]
ZIF-67	42.25 (0.96)	41.37 (0.94)[25]
Zr-fumarate	79.22 (1.80)	80.10 (1.82)[26]
ZIF-90	104.30 (2.37)	---
MIL-53(Al)	98.6 (2.24)	110.03 (2.50)[27]
CAU-10-H	98.60 (2.24)	110.20 (2.30)[28]
**Zeolites**		
Zeolite (Sigma)	134.23 (3.05)*	---
Zeolite 5A	47.97 (1.09)*	---
Zeolite 13X	69.54 (1.58)*	---
Zeolite RhO	115.7 (2.63)	154.04 (3.50)[29]
* Used directly from supplier.		

CO_2 uptake was measured for each of the dispersions as given in Tables 1a-1b. The "predicted" (i.e ideal) uptake was calculated using simple weighted contributions according to the composition of the porous liquid, the gas solubility in the pure liquid (M_{liquid}) component and the uptake of the pure solid (M_{solid}) as in **eq. 1**.

$$\text{Calculated uptake values: } M_{solid} \times w_{solid} + M_{liquid} \times w_{liquid} \qquad \textbf{eq. 1}$$

where w_{solid} and w_{liquid} are the weight fractions of the solid and the liquid in the porous liquid. All solid content in porous liquids are measured at w_{solid} = 12.5 wt% loading, unless specified.

A direct comparison of the measured and predicted (eq. 1) gas uptake values (Δ = experimental - predicted uptake values), shown in Table **S4a - c** below, allows us to infer whether or not the liquid phase has entered the pores of the solid.

Table S4a: CO_2 uptake of porous liquids based on silicone oil, fluorinated oils and paraffin oil (mmol/g)

		Silicone oil 20cst			silicone oil 50cst			silicone oil 350cst			silicone oil 1000cst			Silicone based oil AR20			Fomblin Y oil 60cst			Krytox oil 177 cst			Paraffin oil		
		0.08			0.11			0.09			0.07			0.07			0.07			0.09			0.08		
		exp.	cal.	Δ	exp.	cal.	Δ	exp.	cal.	Δ	exp.	cal.	Δ	exp.	cal.	Δ	exp.	cal.	Δ	exp.	cal.	Δ	exp.	cal.	Δ
**MOFs**																									
HKUST-1	3.88	-	-	-	0.54	0.56	-0.02	0.56	0.56	0.00	0.52	0.56	-0.04	0.56	0.56	0.00	0.14	0.33	-0.19	0.17	0.34	-0.12	0.07	0.56	-0.35
ZIF-8	0.84	0.20	0.18	0.02	0.22	0.22	0.00	0.20	0.19	0.01	0.18	0.18	0.00	-	-	-	0.15	0.12	0.03	-	-	-	0.19	0.18	0.01
Al(fum)(OH)	2.17	-	-	-	0.36	0.37	-0.01	0.38	0.35	0.03	0.38	0.34	0.04	-	-	-	0.26	0.22	0.04	-	-	-	0.31	0.36	-0.05
SIFSIX-3-Zn	2.70	-	-	-	0.45	0.48	-0.03	0.41	0.41	0.00	0.38	0.41	-0.03	0.44	0.41	0.03	0.19	0.26	-0.07	-	-	-	0.39	0.43	-0.04
SIFSIX-3-Cu	2.48	0.34	0.38	-0.04	0.37	0.41	-0.04	-	-	-	-	-	-	0.40	0.38	0.02	0.23	0.24	-0.01	0.22	0.27	-0.05	0.36	0.36	0.00
UiO-66	1.80	-	-	-	0.26	0.33	-0.07	-	-	-	-	-	-	0.31	0.29	0.02	-	-	-	-	-	-	0.34	0.30	0.04
UiO-66-NH2	2.06	-	-	-	0.35	0.36	-0.01	-	-	-	-	-	-	0.31	0.31	0.01	-	-	-	-	-	-	-	-	-
ZIF-67	0.96	-	-	-	0.24	0.22	0.02	-	-	-	-	-	-	-	-	-	-	-	-	-	-	-	-	-	-
Zr-fumarate	1.80	-	-	-	0.38	0.33	0.05	-	-	-	-	-	-	-	-	-	-	-	-	-	-	-	-	-	-
ZIF-90	2.37	-	-	-	0.36	0.40	-0.04	-	-	-	-	-	-	-	-	-	-	-	-	-	-	-	-	-	-
MIL-53(Al)	2.24	-	-	-	0.71	0.64	0.07	-	-	-	-	-	-	-	-	-	-	-	-	-	-	-	-	-	-
CAU-10-H	2.24	-	-	-	0.39	0.38	0.01	-	-	-	-	-	-	-	-	-	-	-	-	-	-	-	-	-	-
CD-MOF-1	0.89	-	-	-	-	-	-	-	-	-	-	-	-	-	-	-	-	-	-	-	-	-	-	-	-
**Zeolite**																									
Zeolite (Sigma)	3.05	0.52	0.45	0.05	0.46	0.47	-0.01	-	-	-	-	-	-	-	-	-	-	-	-	-	-	-	0.46	0.46	0.00
Zeolite 5A	1.09				0.22	0.22	0.00	-	-	-	-	-	-	-	-	-	-	-	-	-	-	-	0.21	0.21	0.00
Zeolite 13X	1.58				0.31	0.30	0.01	-	-	-	-	-	-	-	-	-	-	-	-	-	-	-	0.31	0.30	0.01
**COFs**																									
PAF-1*	4.17	-	-	-	0.23	0.24	-0.01	-	-	-	-	-	-	-	-	-	-	-	-	-	-	-	0.21	0.21	0.00

*3 wt% solid loading; Δ represents difference between experimental and predicted values

Table S4b: CO_2 uptake of porous liquids based on triglyceride oils (mmol/g)

		Brominated vegetable oil			Olive oil			Castor oil			sesame oil			sunflower oil			safflower oil			soy bean oil			corn oil		
		0.10			0.09			0.07			0.08			0.08			0.08			0.07			0.09		
		exp.	cal.	Δ	exp.	cal.	Δ	exp.	cal.	Δ	exp.	cal.	Δ	exp.	cal.	Δ	exp.	cal.	Δ	exp.	cal.	Δ	exp.	cal.	Δ
**MOFs**																									
HKUST-1	3.88	0.56	0.58	-0.02	0.57	0.56	0.01	0.53	0.58	-0.05	0.54	0.57	-0.03	0.57	0.58	-0.01	0.58	0.57	0.01	0.54	0.57	-0.03	0.58	0.58	0.00
ZIF-8	0.84	0.17	0.20	-0.03	0.21	0.18	0.03	0.19	0.18	0.01	0.20	0.18	0.02	0.18	0.17	0.01	0.18	0.17	0.01	0.18	0.18	0.00	0.20	0.18	0.02
Al(fum)(OH)	2.17	0.37	0.36	0.01	0.35	0.34	0.01	0.32	0.35	-0.03	0.36	0.35	0.01	0.35	0.36	-0.01	0.36	0.36	0.00	0.34	0.34	0.00	0.35	0.35	0.00
SIFSIX-3-Zn	2.70	-	-	-	0.39	0.40	-0.01	0.39	0.39	0.00	0.39	0.39	0.00	0.39	0.39	0.00	0.40	0.39	0.01	0.39	0.39	0.00	0.40	0.39	0.01
SIFSIX-3-Cu	2.48	-	-	-	0.35	0.36	-0.01	0.35	0.35	0.00	0.35	0.35	0.00	0.35	0.35	0.00	0.35	0.35	0.00	0.35	0.36	-0.01	0.35	0.35	0.00
UiO-66	1.80	-	-	-	0.26	0.30	-0.04	-	-	-	0.30	0.31	-0.01	-	-	-	-	-	-	-	-	-	-	-	-
UiO-66-NH2	2.06	-	-	-	0.35	0.33	0.02	-	-	-	-	-	-	-	-	-	-	-	-	-	-	-	-	-	-
ZIF-67	0.96	-	-	-	0.19	0.18	0.01	-	-	-	-	-	-	-	-	-	-	-	-	-	-	-	-	-	-
Zr-fumarate	1.80	-	-	-	0.24	0.29	-0.05	-	-	-	-	-	-	-	-	-	-	-	-	-	-	-	-	-	-
ZIF-90	2.37	-	-	-	0.33	0.36	-0.03	-	-	-	-	-	-	-	-	-	-	-	-	-	-	-	-	-	-
MIL-53(Al)	2.24	-	-	-				-	-	-	-	-	-	-	-	-	-	-	-	-	-	-	-	-	-
CAU-10-H	2.24	-	-	-	0.32	0.34	-0.02	-	-	-	-	-	-	-	-	-	-	-	-	-	-	-	-	-	-
CD-MOF-1	0.89	-	-	-	0.18	0.18	0.00	-	-	-	-	-	-	-	-	-	-	-	-	-	-	-	-	-	-
**Zeolite**																									
Zeolite (Sigma)	3.05	-	-	-	0.47	0.48	-0.01	0.47	0.47	0.00	0.46	0.48	-0.02	0.46	0.47	-0.01	0.46	0.46	0.00	0.46	0.46	0.00	0.46	0.46	0.00
Zeolite 5A	1.09	-	-	-	0.22	0.21	0.01	0.22	0.22	0.00	0.21	0.22	-0.01	0.21	0.22	-0.00	0.21	0.22	-0.01	0.21	0.22	-0.01	0.22	0.22	0.00
Zeolite 13X	1.58	-	-	-	0.31	0.30	0.01	0.31	0.31	0.00	0.30	0.31	-0.01	0.31	0.30	0.01	0.31	0.31	0.00	0.32	0.32	0.00	0.31	0.31	0.00
**COFs**																									
PAF-1*	4.17	-	-	-	0.23	0.21	0.02	0.20	0.19	0.01	0.21	0.20	0.01	0.19	0.20	-0.01	0.21	0.21	0.00	0.19	0.19	0.00	0.21	0.21	0.00

*3 wt% solid loading; Δ represents difference between experimental and predicted values

Table S4c: CO_2 uptake of porous liquids based on polyethylene glycol (mmol/g)

		Genosorb® 1753			Polypropylene glycol			Poly(ethylene glycol) PEG-200			polyethylene bis(2-ethylhexanoate)			polyethylene glycol dibenzoate			polyethylene dimethyl ether acrylate		
		0.23			0.11			0.19			0.18			0.06			0.1		
		exp.	cal.	Δ	exp.	cal.	Δ	exp.	cal.	Δ	exp.	cal.	Δ	exp.	cal.	Δ	exp.	cal.	Δ
**MOFs**																			
HKUST-1	3.88	0.08	0.69	-0.43	-	-	-	-	-	-	-	-	-	-	-	-	-	-	-
ZIF-8	0.84	0.13	0.30	-0.17	0.09	0.21	-0.12	0.01	0.66	-0.65	0.26	0.26	0.00	0.15	0.16	-0.01	0.17	0.20	-0.03
Al(fum)(OH)	2.17	0.27	0.46	-0.19	-	-	-	0.16	0.28	-0.12	0.13	0.43	-0.30	0.10	0.33	-0.23	-	-	-
SIFSIX-3-Zn	2.70	-	-	-	-	-	-	-	-	-	-	-	-	-	-	-	-	-	-
SIFSIX-3-Cu	2.48	-	-	-	-	-	-	-	-	-	-	-	-	-	-	-	-	-	-
UiO-66	1.80	-	-	-	-	-	-	-	-	-	-	-	-	-	-	-	-	-	-
UiO-66-NH2	2.06	-	-	-	-	-	-	-	-	-	-	-	-	-	-	-	-	-	-
ZIF-67	0.96	-	-	-	-	-	-	-	-	-	-	-	-	-	-	-	-	-	-
Zr-fumarate	1.80	-	-	-	-	-	-	-	-	-	-	-	-	-	-	-	-	-	-
ZIF-90	2.37	-	-	-	-	-	-	-	-	-	-	-	-	-	-	-	-	-	-
MIL-53(Al)	2.24	-	-	-	-	-	-	-	-	-	-	-	-	-	-	-	-	-	-
CAU-10-H	2.24	-	-	-	-	-	-	-	-	-	-	-	-	-	-	-	-	-	-
CD-MOF-1	0.89	-	-	-	-	-	-	-	-	-	-	-	-	-	-	-	-	-	-
**Zeolite**																			
Zeolite (Sigma)	3.05	0.14	0.60	-0.44	-	-	-	-	-	-	0.09	0.54	0.32	-	-	-	-	-	-
Zeolite 5A	1.09	0.01	0.34	-0.33	-	-	-	-	-	-	-	-	-	-	-	-	-	-	-
Zeolite 13X	1.58	0.03	0.44	-0.41	-	-	-	-	-	-	-	-	-	-	-	-	-	-	-
**COFs**																			
PAF-1	4.17	0.73	0.73	0.00	-	-	-	-	-	-	-	-	-	-	-	-	-	-	-

*3 wt% solid loading; Δ represents difference between experimental and predicted values

As shown in Tables 1a and 1b, most of the compositions show enhanced CO_2 uptake compared to the pure oils and the experimental values are comparable to the values predicted from the uptakes and proportions of the components using eq. 1, indicating that CO_2 uptake behaviour of porous liquids seems to be nearly ideal and thus predictable.

In cases where highly thermally stable liquid media were used (e.g. silicone oils), T3PLs could be activated directly without pre-activation of porous solids before forming T3PLs.

Table S4d: CO_2 uptake of pre-activated vs post-activated porous liquids (mmol/g)

	Pre-activated solid	Activation in oil
Al(fum)(OH)/PDMS	0.43 (200°C, 2h)	0.41 (200°C, 2h)
SIFSIX-3-Zn/PDMS	0.45 (55°C, 3h)	0.45 (85°C, 3h)

Additionally, higher (25wt%) loadings were analysed for some solid/liquid combinations and good agreement between experimental and predicted uptakes were again seen (**Table S4e**).

Table S4e: CO_2 uptake of porous liquids with 25wt% porous solid loading (mmol/g)

	**Experimental**	**Predicted**
ZIF-8 in sesame oil	0.25	0.27
HKUST-1 in silicone oil	0.97	1.00
Zn-SIFSIX-3 in paraffin oil	0.62	0.60
Al(fum)(OH) in olive oil	0.64	0.60

CH_4 and N_2 uptake

Table S5a: CH_4 uptake of porous liquids (mmol/g)

		Silicone oil 50cst			Paraffin oil			Olive oil			Castor oil			Sesame oil		
		0.08			0.07			0.08			0.07			0.08		
		exp.	cal.	Δ	exp.	cal.	Δ	exp.	cal.	Δ	exp.	cal.	Δ	exp.	cal.	Δ
**MOFs**																
HKUST-1	0.96	0.17	0.19	-0.02	0.07	0.13	-0.06	0.18	0.20	-0.02	0.20	0.19	0.01	0.19	0.20	-0.01
ZIF-8	0.71	0.15	0.16	-0.01	0.12	0.12	0.00	0.15	0.16	-0.01	0.16	0.16	0.00	0.16	0.16	0.00
Al(fum)(OH)	1.21	0.21	0.22	-0.01	0.15	0.13	0.02	0.21	0.23	-0.02	0.23	0.22	0.01	0.25	0.23	0.02
SIFSIX-3-Zn	0.97	0.18	0.19	-0.01	0.14	0.13	0.01	0.22	0.19	0.03	0.18	0.19	-0.01	0.21	0.20	0.01
SIFSIX-3-Cu	1.01	0.20	0.19	0.01	0.14	0.13	0.01	0.20	0.20	0.00	0.18	0.19	-0.01	0.21	0.20	0.01
**Zeolite**																
Zeolite (Sigma)	0.08	0.08	0.08	0.00	0.07	0.07	0.00	0.09	0.08	0.01	0.08	0.08	0.00	0.10	0.08	0.02
Zeolite 5A	0.45	0.12	0.12	0.00	0.09	0.08	0.01	0.10	0.12	-0.02	0.09	0.08	0.01	0.09	0.12	-0.03
Zeolite 13X	0.33	0.10	0.11	-0.01	0.08	0.08	0.00	0.06	0.11	-0.05	0.07	0.10	-0.03	0.10	0.11	-0.01
**COFs**																
PAF-1*	1.53	0.14	0.11	-0.03	0.10	0.09	0.01	0.12	0.11	0.01	0.11	0.11	0.00	0.10	0.10	0.00

*3 wt% solid loading; Δ represents difference between experimental and predicted values

Table S5a (continued): CH_4 uptake (mmol/g)

		sunflower oil			safflower oil			soy bean oil			corn oil			Genosorb® 1753		
		0.08			0.08			0.09			0.09			0.01		
		exp.	cal.	Δ	exp.	cal.	Δ	exp.	cal.	Δ	exp.	cal.	Δ	exp.	cal.	Δ
**MOFs**																
HKUST-1	0.96	0.20	0.20	0.00	0.20	0.20	0.00	0.20	0.20	0.00	0.20	0.20	0.00	-	-	-
ZIF-8	0.71	0.16	0.16	0.00	0.15	0.16	-0.01	0.16	0.17	-0.01	0.17	0.16	0.01	-	-	-
Al(fum)(OH)	1.21	0.24	0.23	0.01	0.21	0.23	-0.02	0.24	0.24	0.00	0.22	0.24	-0.02	-	-	-
SIFSIX-3-Zn	0.97	0.23	0.19	0.04	0.21	0.19	0.02	0.22	0.20	0.02	0.20	0.20	0.00	-	-	-
SIFSIX-3-Cu	1.01	0.20	0.20	0.00	0.19	0.20	-0.01	0.21	0.20	0.01	0.20	0.20	0.00	-	-	-
**Zeolites**																
Zeolite (Sigma)	0.08	0.08	0.08	0.00	0.08	0.08	0.00	0.08	0.09	-0.01	0.10	0.08	0.02	-	-	-
Zeolite 5A	0.45	0.10	0.12	-0.02	0.08	0.12	-0.04	0.10	0.13	-0.03	0.10	0.13	-0.03	-	-	-
Zeolite 13X	0.33	0.08	0.11	-0.03	0.10	0.11	-0.01	0.10	0.12	-0.02	0.10	0.12	-0.02	-	-	-
**COFs**																
PAF-1	1.53	*0.13	0.10	0.03	*0.11	0.11	0.01	*0.11	0.12	-0.02	*0.12	0.12	0.00	0.14	0.13	0.00

*3 wt% solid loading; Δ represents difference between experimental and predicted values

Table S6a: N_2 uptake of porous liquids (units: mmol/g)

		silicone oil 50cst			Paraffin oil			Olive oil			Castor oil			sesame oil		
		0.06			0.09			0.08			0.07			0.09		
		exp.	cal.	Δ	exp.	cal.	Δ	exp.	cal.	Δ	exp.	cal.	Δ	exp.	cal.	Δ
**MOFs**																
HKUST-1	0.45	0.11	0.11	0.00	-	-	-	0.20	0.20	0.00	0.12	0.18	-0.06	0.18	0.19	-0.01
ZIF-8	0.21	0.09	0.10	-0.01	0.06	0.10	-0.04	0.07	0.09	-0.02	0.06	0.08	-0.02	0.07	0.10	-0.03
Al(fum)(OH)	0.24	0.09	0.09	0.00	0.11	0.11	0.00	0.10	0.11	-0.01	0.10	0.11	-0.01	0.11	0.12	-0.01
SIFSIX-3-Zn	0.31	0.09	0.09	0.00	0.11	0.11	0.00	0.11	0.12	-0.01	0.10	0.11	-0.01	0.12	0.13	-0.01
SIFSIX-3-Cu	0.37	0.09	0.10	-0.01	0.09	0.12	-0.03	0.08	0.11	-0.03	0.08	0.10	-0.02	0.09	0.12	-0.03
**Zeolites**																
Zeolite (Sigma)	0.58	0.12	0.13	-0.01	0.08	0.15	-0.07	0.12	0.14	-0.02	0.10	0.13	-0.03	0.12	0.15	-0.03
Zeolite 5A	0.46	0.11	0.11	0.00	0.19	0.20	-0.01	0.20	0.20	0.00	0.16	0.18	-0.02	0.18	0.20	-0.02
Zeolite 13X	0.61	0.12	0.13	-0.01	0.09	0.15	-0.06	0.12	0.13	-0.01	0.13	0.13	0.00	0.14	0.13	0.01
**COFs**																
PAF-1*	0.57	0.09	0.08	0.01	0.09	0.08	0.01	0.09	0.10	-0.01	0.07	0.08	-0.01	0.11	0.11	0.00
*3 wt% solid loading; Δ represents difference between experimental and predicted values																

Table S6a (continued): N_2 uptake of porous liquids (units: mmol/g)

		sunflower oil			safflower oil			soy bean oil			corn oil			Genosorb® 1753		
		0.09			0.09			0.09			0.09			0.01		
		exp.	cal.	Δ	exp.	cal.	Δ	exp.	cal.	Δ	exp.	cal.	Δ	exp.	cal.	Δ
**MOFs**																
HKUST-1	0.45	0.18	0.20	-0.02	0.21	0.20	0.01	0.20	0.20	0.00	0.19	0.20	-0.01			
ZIF-8	0.21	0.07	0.10	-0.03	0.07	0.10	-0.03	0.07	0.10	-0.03	0.08	0.10	-0.02			
Al(fum)(OH)	0.24	0.11	0.11	0.00	0.12	0.11	0.01	0.11	0.12	-0.01	0.11	0.12	-0.01			
SIFSIX-3-Zn	0.31	0.11	0.12	-0.01	0.12	0.12	0.00	0.11	0.13	-0.02	0.14	0.12	0.02			
SIFSIX-3-Cu	0.37	0.09	0.12	-0.03	0.09	0.12	-0.03	0.09	0.12	-0.03	0.09	0.12	-0.03			
**Zeolite**																
Zeolite (Sigma)	0.58	0.11	0.15	-0.04	0.11	0.15	-0.04	0.12	0.15	-0.03	0.12	0.15	-0.03			
Zeolite 5A	0.46	0.18	0.20	-0.02	0.18	0.20	-0.02	0.20	0.20	0.00	0.19	0.20	-0.01			
Zeolite 13X	0.61	0.13	0.13	0.00	0.12	0.13	-0.01	0.14	0.13	0.01	0.13	0.13	0.00			
**COFs**																
PAF-1	0.57	*0.13	0.13	0.00	*0.11	0.10	0.01	*0.10	0.11	-0.01	*0.09	0.10	-0.01	0.03	0.07	-0.04
*3 wt% solid loading; Δ represents difference between experimental and predicted values																

CO_2 uptakes versus CH_4 and N_2 uptakes are expressed by the ratio ($A_{mmol/g}/B_{mmol/g}$). Representative examples for CO_2 selectivity over N_2 (CO_2/N_2) and over CH_4 (CO_2/CH_4) were calculated for the porous liquids and the results are shown in **Tables S7a and S7b**.

Table S7a: CO_2 capacity over CH_4 (CO_2/CH_4)

	silicone oil 50cst		Paraffin oil		Olive oil	
	1.38		1.14		1.13	
	exp.	cal.	exp.	cal.	exp.	cal.
**MOFs**						
HKUST-1	3.11	3.07			3.15	2.84
ZIF-8	1.46	1.34	1.58	1.53	1.43	1.13
Al(fum)(OH)	1.69	1.68	2.08	2.68	1.64	1.45
SIFSIX-3-Zn	2.48	2.47	2.44	3.40	1.81	2.05
SIFSIX-3-Cu	1.69	2.12	2.52	2.83	1.74	1.84
**Zeolites**						
Zeolite (Sigma)	5.87	5.76	6.39	6.74	5.35	5.82
Zeolite 5A	1.85	1.83	2.43	2.58	2.26	1.77
Zeolite 13X	3.04	2.72	3.80	3.84	4.86	2.76
**COFs**						
PAF-1	1.64	2.19	2.10	2.33	1.92	2.18

Table S7b: CO_2 capacity over N_2 (CO_2/N_2)

	silicone oil 50cst		Paraffin oil		Olive oil	
	1.83		0.88		1.13	
	exp.	cal.	exp.	cal.	exp.	cal.
**MOFs**						
HKUST-1	3.89	4.85	-	-	2.79	2.83
ZIF-8	3.63	2.68	2.88	1.79	3.16	2.01
Al(fum)(OH)	7.12	4.67	2.96	3.15	3.46	3.05
SIFSIX-3-Zn	6.26	6.21	3.21	3.73	3.47	3.28
SIFSIX-3-Cu	4.58	4.58	4.08	3.01	4.22	3.27
**Zeolites**						
Zeolite (Sigma)	3.94	4.19	5.55	3.06	4.09	3.40
Zeolite 5A	1.95	1.82	1.15	1.07	1.07	1.06
Zeolite 13X	2.57	2.36	3.58	2.01	2.63	2.35
**COFs**						
PAF-1	2.56	3.00	2.56	2.10	1.50	1.62

Most of the compositions exhibit greater selectivity for CO_2 over N_2 and CH_4 than do the pure oils, indicating the addition of porous solid enhances the overall CO_2 selectivity over to N_2 and CH_4.

S.I. 7: CO_2 uptake kinetic data for selected T3PLs

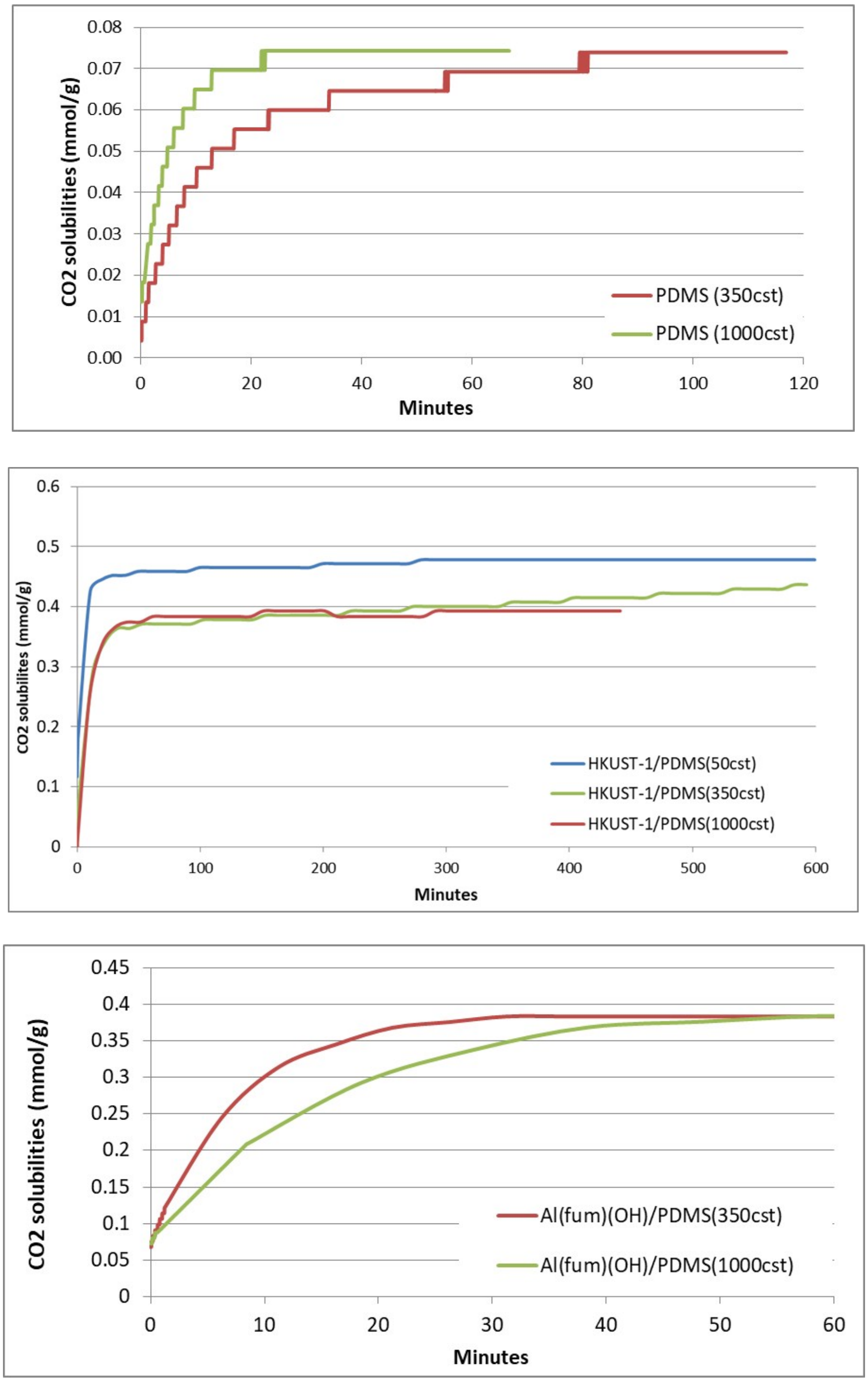

Figure S16: CO_2 uptake versus time for selected T3PLs for PDMS (350 cst and 1000 cst) (top); HKUST-1/PDMS (50 cst, 350 cst and 1000 cst) (middle) and Al(fum)(OH)/PDMS (350 cst and 1000 cst) (bottom)

S.I. 8 PALS measurements

Positron annihilation lifetime spectroscopy (PALS) is now a well-established and efficient probe for detecting and, when possible, quantifying open volumes (pores) in solid molecular media. In this experimental technique[30-32], a positron (from a radioactive ^{22}Na source) with a moderate energy spread (up to a few hundred keV) is injected into such media, it quickly loses its energy and comes into thermal equilibrium with the host matrix (energy ~ $k_B T$; k_B is the Boltzmann constant and *T* is the temperature of the host matrix). The thermalized positron will eventually annihilate with an electron of the medium. It can annihilate as a free particle with an electron of the medium into two γ-photons each carrying the mass-energy (~511 keV) of the annihilating particles. However, due to energy advantage, a significant fraction of the thermalized positrons forms a bound state, positronium (Ps), a hydrogen-like atom consisting of the positron and an electron of the medium prior to annihilation. Due to the relatively large size of the Ps atoms, they only form within and annihilate from the pores in molecular media, thus making them unique and efficient probes of such 'holes' in the matrix. Depending on the spin configurations of the two particles, a Ps atom form as a *para*-positronium (p-Ps: anti-parallel positron and electron spins; total spin *0*) or an *ortho*-Positronium (o-Ps: parallel positron and electron spins; total spin *1*). The total spins of the two Ps-states dictate that an o-Ps has an abundance of three times that of p-Ps. In vacuum, a p-Ps annihilates into two γ-photons with a lifetime of 125 ps while an o-Ps annihilates into three γ-photons with a lifetime of 142 ns which is three orders of magnitude larger. During the relatively long lifetime of the more abundant o-Ps, it undergoes numerous collisions with the surrounding walls of the pores. During these collisions, the positron has the propensity to annihilate with an electron from the pore wall and of opposite spin because this mode of annihilation is quantum mechanically more probable. This so-called 'o-Ps pick-off annihilation' will result in the emission of two (rather than three) γ-photons and a drastically reduced o-Ps lifetime (a few ns compared to vacuum lifetime of ~142 ns) [30-32]. The magnitude of this reduction in lifetime is governed by the number of collisions of the o-Ps with the 'pore-walls', thus providing a relatively accurate correlation to the pore sizes and size distributions[30-32]. In the same fashion, the presence of any ingressing molecules into the pores, the collisions of the o-Ps with molecules within the pore would also reduce the o-Ps lifetime providing a signature of the changed pore geometry. [30-32]

A measured positron lifetime spectrum would thus consist of multiple components (as can be seen in **Figure S17**) arising from annihilations of free positrons, p-Ps, o-Ps and, in more complex situations, other annihilation modes. Given the possibility of a large dispersion in pore sizes in molecular media, there may be a large spread of the o-Ps lifetime values. Therefore, in the analysis of the lifetime spectra, we use fitting of both discrete lifetimes of each annihilation modes as well as lifetime distributions around the individual discrete values. [30-32] In The lifetime distribution mode, the 'peak' of the distribution gives a measure of the average lifetime obtained from the discrete mode. In the context of this paper, it is the o-Ps lifetimes that provide the information regarding the pore sizes. In complex situations, as is the case here, evaluating a combination of the average lifetimes and their spread are essential to arrive at a comprehensive picture of the underlying physics/chemistry.

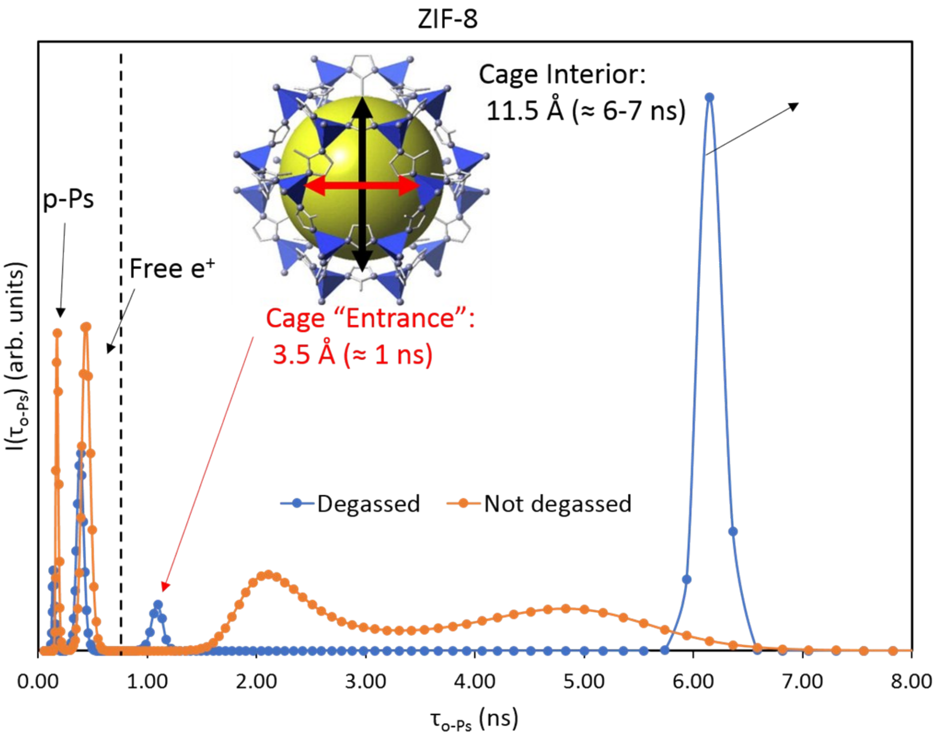

Figure S17: O-Ps lifetime distribution of ZIF-8 (values on the RHS of the black dotted vertical line) without the presence of gas (blue) and with the ingress of gas into the pores. For illustration purposes, the p-Ps and free positron lifetimes (LHS of the dotted vertical line) are also included. The insert at the top shows schematic description of ZIF-8 cage.

To illustrate the principles, we first look at a detailed lifetime analysis for pure ZIF-8 in **Figure S17**. Here, the samples were measured as received (not degassed) and then the gas was removed from the pores by pumping on the sample in-situ over a few days until there was no change in the measured spectra indicating close to complete degassing of sample. In the absence of gas in the pores, we obtain two o-Ps lifetimes of ~1 ns and ~6 ns, both with narrow distributions indicating the existence of relatively well-defined ‘pores‘ of average diameters of ~3.5 and 11.5 Å. We interpret the shorter of the two lifetimes as the signature of the cage aperture of ZIF-8 and the larger lifetime as the signature of cage interiors in ZIF-8. In the presence of gas ingressed into the pores from the atmosphere, we observe a more complex picture. There is a reduction of the o-Ps ~6 ns lifetime to a broader lifetime distribution extending between 3 to 6 ns reflecting the pick-off annihilation characteristics in the presence of ingressed molecules. A second observed broad distribution around 2 ns is likely to be due to the formation of adsorbed layers of gas on to the pore surfaces reducing the dimensions of some of the pores. The presence of a multitude of annihilation components also tends to result in merger of closely situated components in the analysis process which is evident here in the merging of the 1 ns component in the gas free ZIF-8 into this new ~2 ns component. This example illustrates that, even if a precise quantitative evaluation of pore-dimensions in such complex situations is not feasible, the lifetime analysis can elucidate the changes in relative dimensions and the underlying mechanisms even in complex situations.

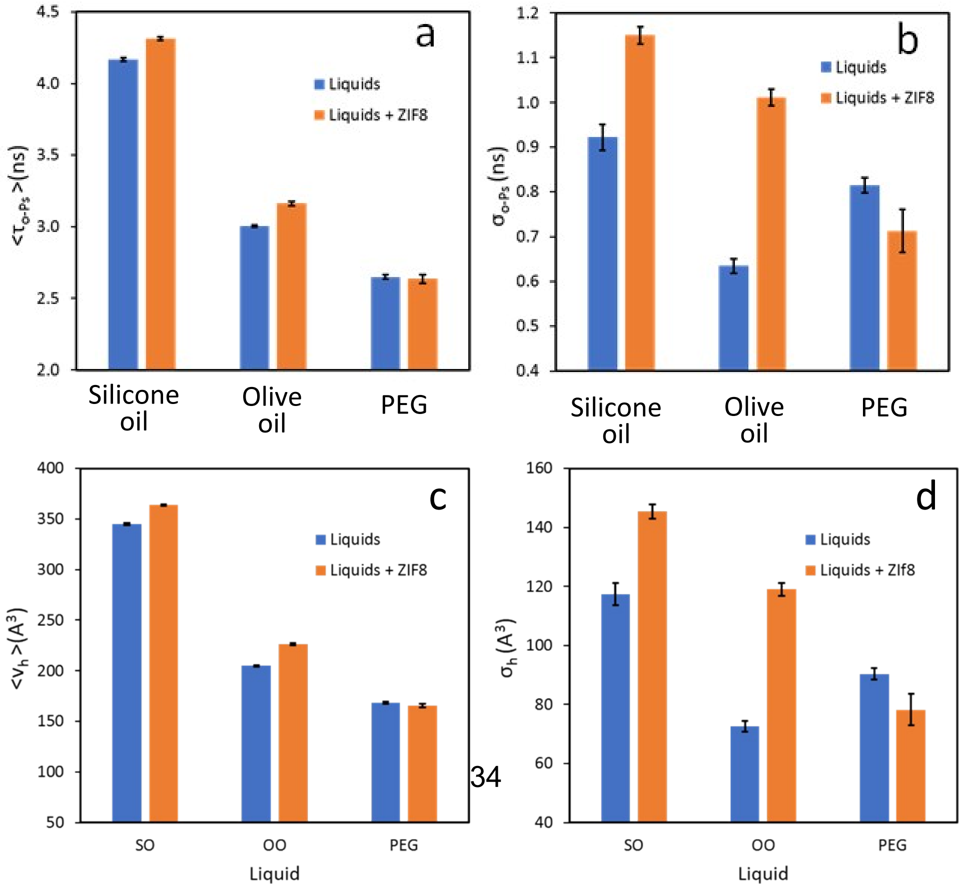

Silicone oil | Olive oil | PEG | Silicone oil | Olive oil | PEG

Figure S18: o-Ps average lifetimes (+lifetime distributions) and free-volume hole sizes (+ distributions) from PALS of pure and porous liquids

In liquids with no pre-existing pores, a positronium can localize itself in a so called positronium 'bubble' which is an empty volume (pore) created within the liquid by the positronium itself by dissipating its zero point energy which is balanced by the surface tension of the inner surface of the bubble created. Thus, the created pore dimension is characteristic of the liquid. In the case of the pure liquids, for example for silicon oil, we observe an o-Ps lifetime of τ_{o-Ps}≈4.167 ±0.13 ns (**Figure S18**) with a lifetime distribution of σ_{o-Ps} ≈0.9±0.023 ns (Silicone oil, **Figure S18**). The corresponding average size of the bubble of v_h~345 Å^3 with σ_h≈117 Å^3 (**Figure S 18**) is compatible with values expected in silicone oil. The measured o-Ps lifetime values for olive oil and PEG are: τ_{o-Ps}≈3.0 ±0.1 ns (σ_{o-Ps} ≈0.64±0.02 ns) and τ_{o-Ps}≈2.657 ±0.1 ns (σ_{o-Ps} ≈0.82±0.02 ns) respectively. These would give average Ps bubble sizes of ~204 Å^3 (with a spread≈72 Å^3) for silicone oil and ~168 Å^3 (with a spread ≈90 Å^3) for PEG.

With the addition of 12.5 wt% ZIF8 into each liquid, we observe significant changes in the observed o-Ps lifetimes and corresponding pore dimensions compared to the cases of pure liquids. Here, we do not observe shorter second lifetime in the vicinity of 2 ns. In the porous liquid samples consisting of Silicone oil + ZIF-8 and Olive oil + ZIF-8, we observe an appreciable increase in the average pore sizes compared to the pure liquids together with remarkably larger increases in the pore size distributions (see **Figure S18**). The increase in lifetime values reflect a weighted combination of the lifetime ranges of ZIF-8 ingressed with molecules and pure liquids and can only arise from the effects of the addition of the larger pores within the ZIF. The more significantly increased pore size distribution reflects the presence of a wide degree of ingression of liquids into the pores of ZIF-8 and is a confirmation of a larger porosity. For the mixture consisting of PEG + ZIF-8, no increases either in the average pore size or in their distributions are observed. The lack of any effect of the ZIF on the hole sizes here would indicate that the empty volumes of the 'cage-like' structures are more fully penetrated by PEG, eliminating/filling the 'free volumes' within the ZIF. Thus, PALS clearly demonstrates significant overall increases in the 'free-volumes' of the Silicone oil +

ZIF-8 and Olive oil + ZIF-8 porous liquids but not in the case of PEG + ZIF-8. This correlates with our observations of gas uptake in these porous samples.

S.I. 9 Regeneration

Since they are physical sorbents these porous liquids are expected to be easily regenerated by applying mild heating or vacuum. As shown in **Figure S19**, two porous liquids (12.5 wt% HKUST-1/PDMS and 12.5 wt% Al(fum)(OH)/PMDS were found to recover at least 75% of their CO_2 uptake capacity by applying vacuum for 2h. However, the conventional amine-based solution (12.5 wt% MEA/H_2O) shows around 5% recovery.

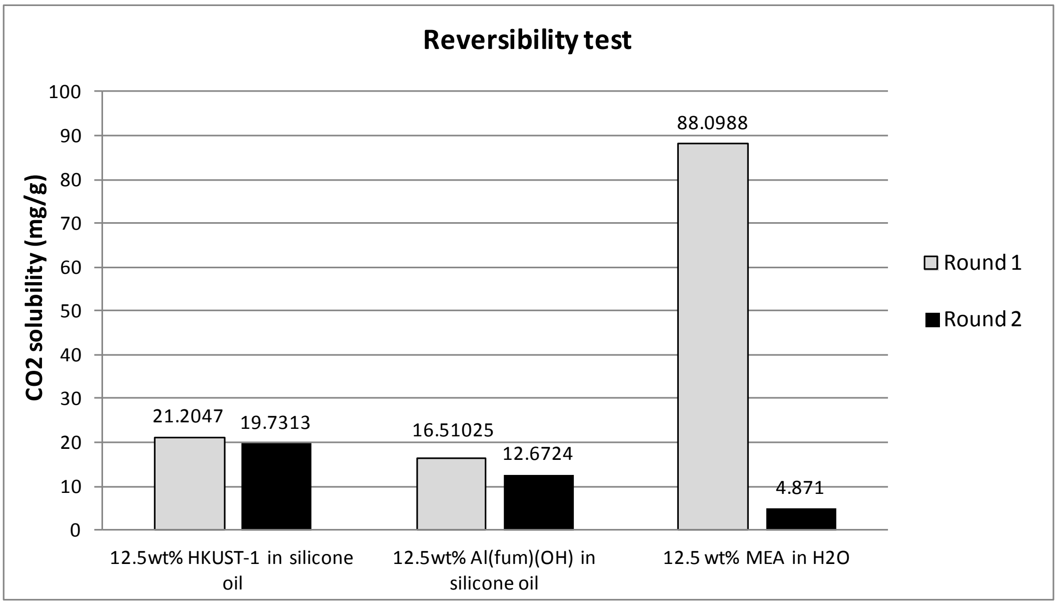

Figure S19: 2 cycles regeneration study of porous liquids (12.5 wt% HKUST-1 in silicone oil and 12.5 wt% Al(fum)(OH) in silicone oil) vs. conventional amine-based solution (12.5 wt% MEA in H_2O).

A regeneration study with HKUST-1/PDMS was also conducted. Preliminary tests using c.a. 13 wt% HKUST-1 in silicone oil shows that almost all of the porous liquid capacity can be regenerated by applying vacuum (up to 8.5 x 10^{-2} bar) to remove captured CO_2 and the CO_2 uptake capacity maintains for at least 5 cycles (**Figure S20**).

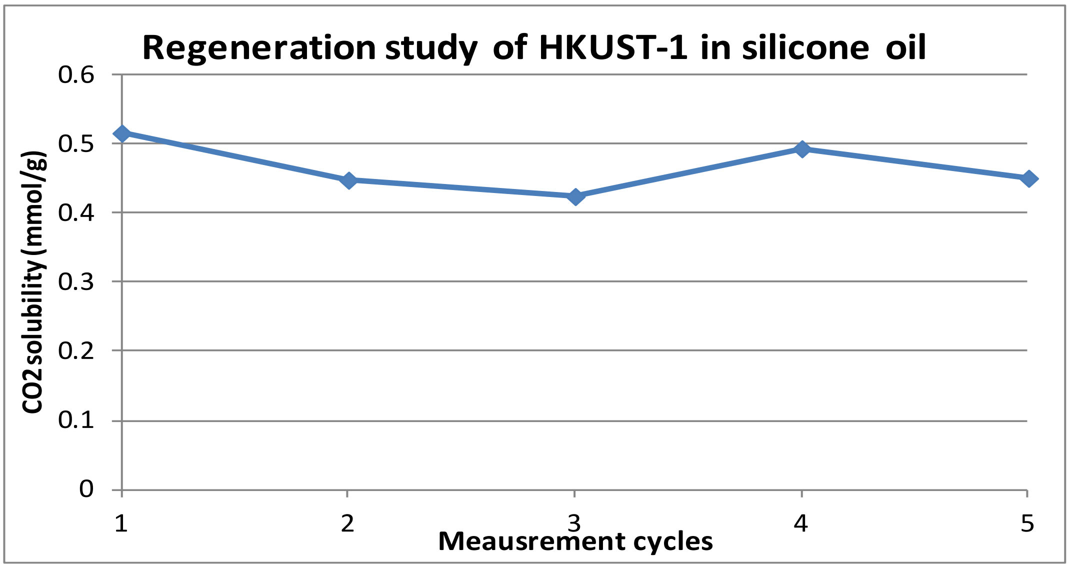

Figure S20: CO_2 solubility in HKUST-1/silicone oil over 5 cycles.

Further regeneration tests for selected porous liquids were conducted at other temperatures (298 K and 323 K) under vacuum for 30 minutes. The results indicated that at least 75% of the porous liquid capacity can be regenerated at 298 K and 100% recovery of porous liquid can be achieved at 323 K (**Figure S21**).

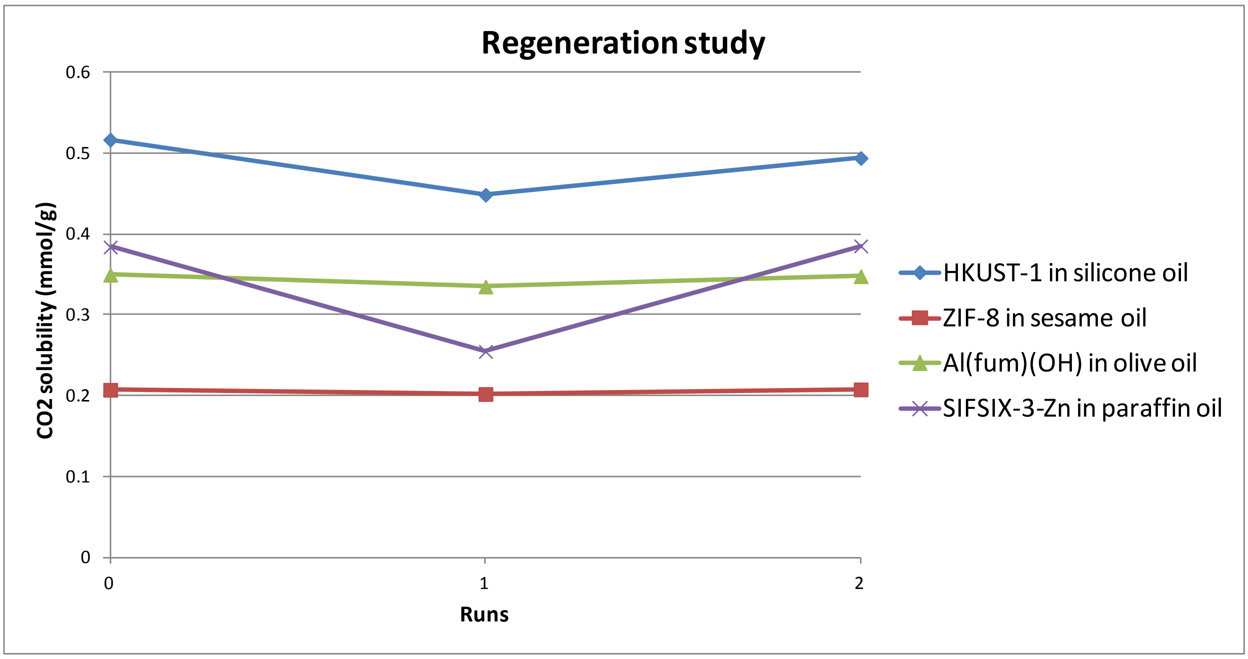

Figure S21: CO_2 solubility of selected porous liquids are shown. Run 0: original CO_2 uptake; Run 0: uptake recorded after regeneration at 298K under vacuum for 30 mins; Run 2: uptake recorded after regeneration at 323K under vacuum for 30 mins.

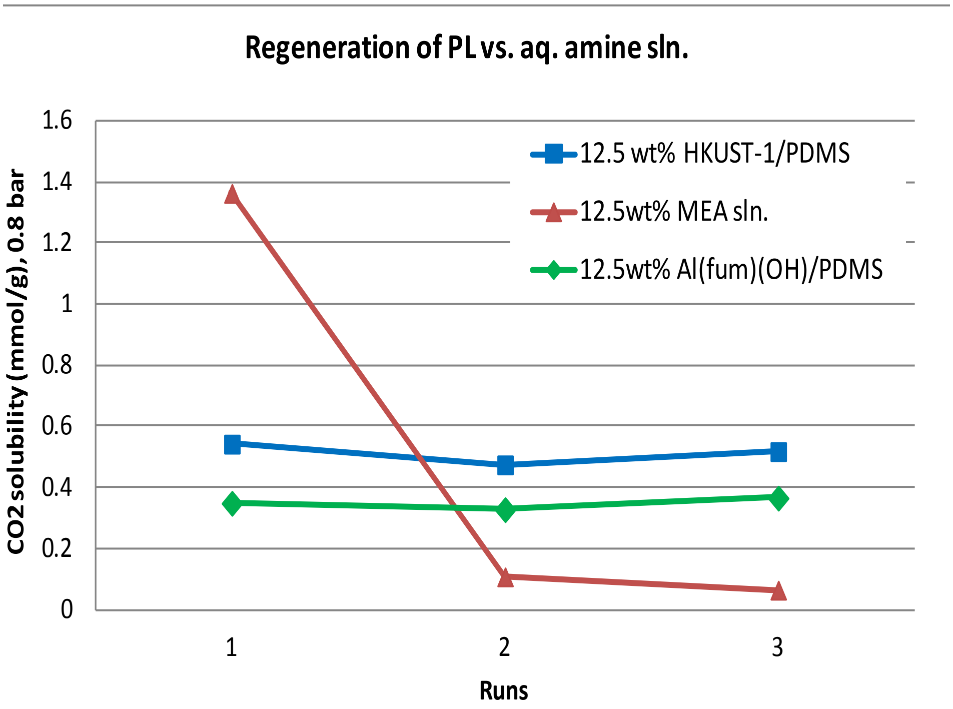

Figure S22: Reproducibility of CO_2 uptake for two Type 3 porous liquids over three cycles using brief evacuation to regenerate the porous liquid between measurements, compared to that of an aqueous amine solution (red) which shows greater initial uptake but is not regenerated under the same conditions

S.I. 10 High pressure gas solubility measurements (1-5 bar, 25°C-75°C)

High pressure gas uptake studies were carried out using a Parr reactor based on mass flow. Data points were taken for 60 minutes at each pressure (**Figure S23**). As shown in the data, at low pressure (1 bar), it takes about 9.3 min for Al(fum)(OH)/PDMS, 3.3 mins for Genosorb® 1753 and 36.4 min for the MEA solution to reach a plateau (saturation). At high pressure (5 bar), it takes ca. 4.6 mins for Al(fum)(OH)/PDMS, 4.6 mins for Genosorb® 1753 and 4.3 min for MEA solution to reach saturation.

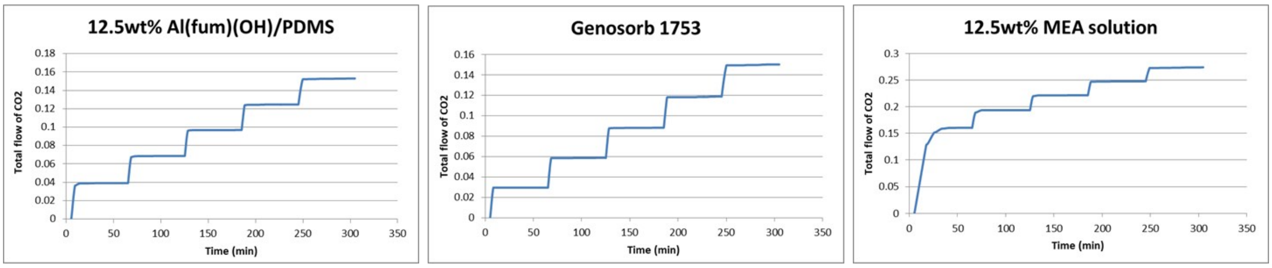

Figure S23: Total flow of CO_2 uptake for 12.5wt% Al(fum)(OH)/PDMS, Genosorb® 1753 and 12.5wt% MEA solution (1 - 5 bar).

All measurements were carried out between 1 - 5 bar at 298 K, 323 K and 348 K. Data for Al(fum)(OH) are given in **Table S8**. As with the low-pressure measurements, the measured CO_2 uptake of porous liquids at high pressure is comparable to the predicted values based on the uptakes of the individual solid and liquid components.

Table S8: Experimental versus predicted CO_2 solubility of 12.5wt% Al(fum)(OH)/PDMS under 1 – 5 bar at 298K, 323K and 348K (mmol/g).

	PDMS			Al(fum)(OH) literature value		
	298K	323K	348K	298K	323K	348K
1 bar	0.104	0.085	0.075	2.1	1.5	0.95
2 bar	0.202	0.165	0.144	3.1	2.2	1.50
3 bar	0.309	0.251	0.226	3.6	2.7	2.00
4 bar	0.420	0.342	0.309	4.0	3.2	2.45
5 bar	0.546	0.439	0.403	4.2	3.5	2.75

	12.5wt% Al(fum)(OH)/PDMS					
	298K		323K		348K	
	Experimental	predicted	Experimental	predicted	Experimental	predicted
1 bar	0.388	0.394	0.268	0.262	0.152	0.184
2 bar	0.564	0.571	0.391	0.419	0.264	0.313
3 bar	0.700	0.714	0.506	0.557	0.355	0.447
4 bar	0.826	0.866	0.613	0.699	0.449	0.577
5 bar	0.971	1.044	0.718	0.822	0.542	0.697

S.I. 11 Ethylene/ethane uptakes

The solubilities of ethylene (C_2H_4) and ethane (C_2H_6) were studied (Table **S9** and **S10**) for selected porous liquids.

Table S9: C_2H_4 solubility data for selected porous liquids (Unit: **mmol/g**).

			Paraffin oil 0.06		Silicone oil 0.10		Sesame oil 0.07	
	Exp.	Lit.	Exp.	Cal.	Exp.	Theo.	Exp.	Theo.
HKUST-1	6.10	---	0.32	0.81	0.74	0.85	0.85	0.83
ZIF-8	2.04	1.39	0.31	0.30	0.33	0.34	0.28	0.32
ZIF-7	1.80	2.10	0.17	0.28	0.35	0.31	0.27	0.20
Zeolite 13X	0.95	---	0.16	0.17	0.16	0.20	0.16	0.18
Zeolite 5A	1.57	2.43	0.30	0.25	0.25	0.28	0.37	0.26
Zeolite AgX	2.98	2.26	0.28	0.42	0.33	0.46	0.29	0.44
Zeolite AgA	1.91	2.10	0.35	0.29	0.45	0.33	0.30	0.30

Table S10: C_2H_6 solubility data for selected porous liquids (Unit: **mmol/g**).

			Paraffin oil 0.13		Silicone oil 0.20		Sesame oil 0.15	
	Exp.	Lit.	Exp.	Cal.	Exp.	Cal.	Exp.	Cal.
HKUST-1	4.19	---	0.20	0.64	0.56	0.67	0.68	0.65
ZIF-8	2.18	2.50	0.41	0.39	0.43	0.44	0.27	0.40
ZIF-7	2.31	2.25	0.45	0.41	0.31	0.46	0.45	0.42
Zeolite 13X	1.72	---	0.35	0.33	0.30	0.39	0.39	0.34
Zeolite 5A	1.08	1.72	0.32	0.25	0.3	0.31	0.33	0.26
Zeolite AgX	1.98	1.45	0.26	0.36	0.40	0.42	0.3	0.38
Zeolite AgA	0.13	0.09	0.08	0.13	0.14	0.19	0.21	0.14

Table S11: Selectivities of porous liquids for C_2H_4 over C_2H_6: (**C_2H_4/C_2H_6**).

		Paraffin oil 0.43		Silicone oil 0.50		Sesame oil 0.50	
		Exp.	Cal.	Exp.	Cal.	Exp.	Cal.
**MOFs**							
HKUST-1	1.45	1.63	1.27	1.32	1.22	1.25	1.27
ZIF-8	0.94	0.77	0.78	0.77	0.77	1.03	0.80
ZIF-7	0.78	0.37	0.68	1.15	0.68	0.58	0.69
**Zeolites**							
Zeolite 13X	0.55	0.45	0.51	0.52	0.53	0.40	0.53
Zeolite 5A	1.46	0.94	0.98	0.84	0.92	1.12	0.99
Zeolite AgX	1.51	1.08	1.16	0.82	1.09	0.80	1.16
Zeolite AgA	15.33	4.49	2.19	3.27	1.74	1.44	2.11

S.I. 12 Biocompatible CD-MOF-1/Olive oil T3PL

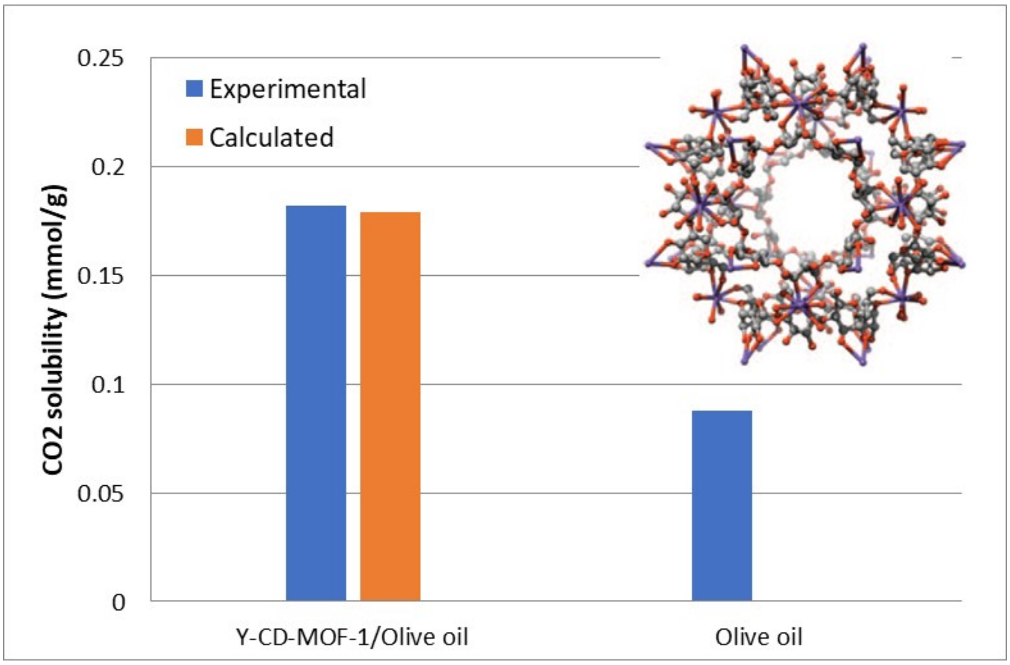

Figure S24: CO_2 uptake of 12.5 wt% CD-MOF-1/olive oil porous liquid versus olive oil.

References

[1] J. Cravillon, R. Nayuk, S. Springer, A. Feldhoff, K. Huber, M. Wiebcke, *Chem. Mater.*, 2011, **23**, 2130.

[2] J-L. Zhuang, D. Ceglarek, S. Pethuraj, A. Terfort, Adv. *Funct. Mater.*, 2011, **21**, 1442.

[3] S. Karmakar, J. Dechnik, C. Janiak, S. De, *Journal of Hazardous Materials*, 2016, **303**, 10.

[4] P. Nugent, Y. Belmabkhout, S. D. Burd, A. J. Cairns, R. Luebke, K. Forrest, T. Pham, S. Ma, B. Space, L. Wojtas, M. Eddaoudi, M. J. Zaworotko, *Nature*, 2013, **493**, 80.

[5] O. Shekhah, Y. Belmabkhout, Z. Chen, V. Guillerm, A. Cairns, K. Adil, M. Eddaoudi, *Nat. Commun.*, 2014, **5,** 4228.

[6] M. J. Katz, Z. J. Brown, Y. J. Colon, P. W. Siu, K.A. Scheidt, R. Q. Snurr, J. T. Hupp, O. K. Farha, *Chem. Commun.* 2013, **49**, 9449.

[7] C. L. Luu, T. T. V. Nguyen, T. Nguyen, T. C. Hoang, *Adv. Nat. Sci: Nanosci. Nanotechnol.*, 2015, **6**, 1.

[8] A. F. Gross, E. Sherman, J. J. Vajo, *Dalton Trans*, 2012, **41**, 5458.

[9] F-K. Shieh, S-C. Wang, S-Y. Leo, K.C.-W. Wu, *Chem. Eur. J.*, 2013, **19**, 11139.

[10] H. Reinsch, S. Waitschat, S. M. Chavan, K. P. Lillerud, N. Stock, *Eur. J. Inorg. Chem.*, 2016, **27**, 4490.

[11] a) W. P. Mounfield, K. S. Walton, *Journal of Colloid and Interface Science*, 2015, **447**, 33; b) A. Taheri, E. G. babakhani, *Adsorption Science & Technology*, 2018, **36**, 247.

[12] H. Reinsch, M. A. van der Veen, B. Gil, B. Marszalek, T. Verbiest, D. de Vos, and N.Stock, *Chemistry of Materials,* 2013, ***25***, 17.

[13] R. A. Smaldone, R. S. Forgan, H. Furukawa, J. J. Gassensmith, A. M. Z. Slawin, O. M. Yaghi, J. F. Soddart, *Angew. Chem. Int. Ed.*, **2010**, 49, 8630.

[14] a) M. Rahimifard, G. M. Ziarani, A. Badiei, F. Yazdian, *J. Inorg. & Organomet. Poly. Mater.*, 2017, **27**, 1037; b) E.S. Sanil, K-H. Cho, D-Y. Hong, J. S. Lee, S. G. Ryu, H. W. Lee, J-S. Chang, Y. K. Hwang, *Chem. Commun.*, 2015, **51**, 8418.

[15] B. Li, Y. Zhang, R. Krishna, K. Yao, Y. Han, Z. Wu, D. Ma, Z. Shi, T. Pham, B. Space, J. Liu, P. K. Thallapally, J. Liu, M. Chrzanowski, S. Ma, *J. Am. Chem. Soc.*, 2014, **136**, 24, 8654

[16] S. Aguado, G. Bergeret, C. Daniel, D. Farrusseng, *J. Am. Chem. Soc.*, 2012, **134**, 14635.

[17] D. S. Jones, Y. Tian, S. Li, T. Yu, O. A. Abu-diak, G. P. Andrews, *J. Pharm. Sci*., 2016, **105,** 3064.

[18] J. Jacquemin, P. Husson, V. Majer, M. F. C. Gomes, *Fluid Phase Equilibria*, 2006, **240**, 87.

[19] P. Aprea, D. Caputo, N. Gargiulo, F. Iucolano, F. Pepe, *J. Chem. Eng. Data*, 2010, **55,** 3655.

[20] S. Gadipelli, W. Travis, W. Zhou, Z. Gou, 2014, *Energy Environ. Sci*., 2014, **7**, 2232.

[21] J. A. Coelho, A. M. Ribeiro, A. F. P. Ferreira, S. M. P. Lucena, A. E. Rodrigues, D. C. S. de Azevedo, *Ind. Eng. Chem. Res*., 2016, **55,** 2134.

[22] X. Cui, K. Chen, H. Xing, Q. Yang, R. Krishna, Z. Bao, H. Wu, W. Zhou, X. Dong, Y. Han, B. Li, Q. Ren, M. J. Zaworolko, B. Chen, *Science*, 2016, **353**, 141.

[23] H. Jasuja, J. Zang, D. S. Sholl, K. S. Walton, *J. Phys. Chem. C*., 2012, **116**, 23526.

[24] C. L. Luu, T. T. V. Nguyen, T. Nguyen, T. C. Hoang, *Adv. Nat. Sci*., 2015, **6,** 25004.

[25] H. Yang, X. He, F. Wang, Y. Kang, J. Zhang, *J. Mater. Chem*., 2012, **22**, 21849.

[26] M. Ganesh, P. Hemalatha, M. M. Peng, W. S. Cha, H. T. Jang, *Aerosol and Air Quality Research*, 2014, **14,** 1605.

[27] P. Mishra, H. P. Uppara, B. Mandal, S. Gumma, *Ind. Eng. Chem. Res*., 2014, **53,** 19747.

[28] H. Reinsch, S Waitschat, N. Stock, *Dalton Transactions*, 2013, **42,** 4840.

[29] M. Palomino, A. Corma, J. L. Jorda, F. Rey, S. Valencia, *Chem. Commun*., 2012, **48,** 215.

[30] D. Kilburn, D); J. Claude, T. Schweizer, M. A. Alam, J. Ubbink, *Biomacromolecules,* 2005, **6,** 864.

[31] D. Bamford, G. Dlubek, G. Dommet, S. Horing, T. Lupke, Kilburn, M.A. Alam, *Polymer,* 2006, **47,** 3486.

[32] D. Hughes, C. Tedeschi, B. Leuenberger, M. Roussenova, A. Coveney, R. Richardson, G. Badolato-Bonisch, M. A. Alam, J. Ubbink, *Food Hydrocolloids*, 2016, **58,** 316.
